# Dynamic Energy Budget models: fertile ground for understanding resource allocation in plants in a changing world

**DOI:** 10.1093/conphys/coac061

**Published:** 2022-09-15

**Authors:** Sabrina E Russo, Glenn Ledder, Erik B Muller, Roger M Nisbet

**Affiliations:** School of Biological Sciences, University of Nebraska, 1104 T Street Lincoln, Nebraska 68588-0118, USA; Center for Plant Science Innovation, University of Nebraska, 1901 Vine Street, N300 Beadle Center, Lincoln, Nebraska 68588-0660, USA; Department of Mathematics, University of Nebraska, 203 Avery Hall, Lincoln, Nebraska 68588-0130, USA; Marine Science Institute, University of California, Santa Barbara, California 93106, USA; Institut für Biologische Analytik und Consulting IBACON GmbH, Arheilger Weg 17 Roß dorf, Hesse D-64380, Germany; Marine Science Institute, University of California, Santa Barbara, California 93106, USA; Department of Ecology, Evolution and Marine Biology, University of California, Santa Barbara, California 93106, USA

## Abstract

Climate change is having dramatic effects on the diversity and distribution of species. Many of these effects are mediated by how an organism’s physiological patterns of resource allocation translate into fitness through effects on growth, survival and reproduction. Empirically, resource allocation is challenging to measure directly and so has often been approached using mathematical models, such as Dynamic Energy Budget (DEB) models. The fact that all plants require a very similar set of exogenous resources, namely light, water and nutrients, integrates well with the DEB framework in which a small number of variables and processes linked through pathways represent an organism’s state as it changes through time. Most DEB theory has been developed in reference to animals and microorganisms. However, terrestrial vascular plants differ from these organisms in fundamental ways that make resource allocation, and the trade-offs and feedbacks arising from it, particularly fundamental to their life histories, but also challenging to represent using existing DEB theory. Here, we describe key features of the anatomy, morphology, physiology, biochemistry, and ecology of terrestrial vascular plants that should be considered in the development of a generic DEB model for plants. We then describe possible approaches to doing so using existing DEB theory and point out features that may require significant development for DEB theory to accommodate them. We end by presenting a generic DEB model for plants that accounts for many of these key features and describing gaps that would need to be addressed for DEB theory to predict the responses of plants to climate change. DEB models offer a powerful and generalizable framework for modelling resource allocation in terrestrial vascular plants, and our review contributes a framework for expansion and development of DEB theory to address how plants respond to anthropogenic change.

## Introduction

Climate change is producing novel environments, creating environment–phenotype mismatches and, as a result, causing rapid changes in species’ habitat and geographic distributions (Williams *et al.*, 2007; Burrows *et al.,* 2014; Smith *et al.*, 2015; Sharma *et al.,* 2022). Understanding the mechanisms underlying species’ distributions is a compelling ecological challenge that has urgent conservation importance if we are to predict species’ responses to a rapidly changing climate. Species distribution models (SDMs) characterize a species’ ecological niche and then map that niche onto environmental data describing future climates to predict species’ geographic range limits and habitat occupancy (Merow *et al.*, 2013). Because many species distribution modelling approaches use contemporary species’ distributions records to infer future range shifts, they lack a mechanistic basis and do not account for an organism’s interactions with its local environment, and therefore have limited predictive capacity in novel environmental settings (Kearney and Porter, 2004; Kearney and Porter, 2009; Kearney *et al.*, 2010; De Frenne *et al.,* 2013; Harwood *et al.*, 2014; Briscoe *et al.,* 2019). Distributions are defined by population dynamic processes involving the birth and death of individuals, which themselves are influenced by fundamental and unavoidable trade-offs in resource allocation to alternative physiological functions, such as between processes affecting growth, defence, storage and reproduction (Detto *et al.*, 2021; Russo *et al.,* 2021). Hence, as a major step towards mechanistic SDMs that link physiology with conservation (Cooke *et al.,* 2013), it is vital to develop generalizable models predicting components of fitness that represent organismal physiology in a way that can account for these trade-offs (Kearney and Porter, 2017; Briscoe *et al.,* 2019), and Dynamic Energy Budgets (DEBs) show promise for these efforts (Lavaud *et al.*, 2021). This is particularly true for plants, as they are notorious for having dramatic phenotypic plasticity driven by ontogeny and environmental conditions (Clausen *et al.*, 1940) that is often mediated by variation in the allocation of endogenous resources to alternative functions (Silvertown *et al.*, 1997). Moreover, unlike mobile animals, plants cannot easily move to find more suitable environments. Developing mechanistic models of resource allocation in plants and plasticity in it is thus fundamental to predicting the responses of plants to climate change (Matesanz *et al.*, 2010; Nicotra *et al.,* 2010; Valladares *et al.,* 2014).

Organisms have finite amounts of resources that they can allocate to different functions. Resource allocation is therefore a zero-sum game, that is, resources allocated to one function are consequently not available to other functions (Stearns, 1992). As a result, unavoidable trade-offs arise that depend on environmental conditions, both abiotic and biotic. However, understanding resource allocation strategies goes beyond the zero-sum analogy, as there are indirect costs and benefits that must be accounted for when considering the consequences of alternative allocation patterns at the level of the whole organism, giving rise to complex feedbacks between allocation and access to resources (Westoby *et al.*, 2000; Ledder *et al.*, 2020). Understanding resource allocation strategies is further complicated by the necessity for phenotypic integration of the whole organism, that is, the functioning of different parts of an organism must be compatible with each other in order to maximize fitness (Pigliucci, 2003). A corollary of phenotypic integration is that there may be different resource allocation strategies that yield the same fitness in a single environment (Marks and Lechowicz, 2006; Worthy *et al.,* 2020), which is consistent with the observation that in nature, multiple resource allocation strategies co-exist in ecological communities (Russo *et al.,* 2021).

Empirically, resource allocation is challenging to measure directly, and so has often been approached using mathematical models invoking principles of economic cost–benefit analysis (Bloom *et al.*, 1985; Ledder *et al.,* 2020). Many organ- and process-specific physiological models exist that predict optimal photosynthetic C-gain for trees and have been used to model niche occupancy of plants (e.g. Sterck *et al.*, 2011; Sterck and Schieving, 2011). While these models represent significant advances, the trade-offs they predict at the whole-plant level do not arise as outcomes of mechanistic representations of resource allocation among organs and physiological processes. Optimality models have commonly been used to model resource allocation in plants, as they invoke the intuitively satisfying assumption that there must be an optimal strategy of allocation for a species, given its life history and environment (Iwasa and Roughgarden, 1984; Bloom *et al.,* 1985; Coley *et al.*, 1985). However, the same feature that makes the optimality approach intuitively satisfying is also a weakness, in that one needs to define a priori the quantity to be maximized, which is usually just one component of fitness, and to provide an objective function that incorporates mechanisms dictating how that optimality is achieved (Ledder *et al.,* 2020). Another possible approach is allowing alternative resource allocation strategies to compete in an evolutionary stable strategy framework, such as in Farrior *et al.* (2013), which examined allocation to above versus belowground structures using an analytically tractable model, an approach that could be applied at larger spatial scales to model species distributions. Semi-mechanistic models based on functional trait variation have also been developed that predict the distribution of plant functional types and implicitly account for trade-offs in that they use empirical distributions of functional traits to describe plant functional types (*e.g.*  Moorcroft *et al.*, 2001; Medvigy *et al.*, 2009) and are the basis for large-scale dynamic global vegetation models (Fisher *et al.,* 2018). Recent work has modelled four empirically well-documented trade-offs in plants related to resource allocation and demonstrated that all of them have the capacity to support long-term coexistence of many species (Detto *et al.,* 2021), demonstrating the importance of trade-offs for modelling plant communities and species distributions.

DEB theory (Kooijman, 1986; Kooijman, 2010) offers another approach to modelling whole organisms, which has a flexible and generalizable framework. Based on first principles of metabolic organization and how energy and mass are allocated to different functions of an organism’s budget, DEB theory is well suited for modelling resource allocation. The DEB modelling approach has been less applied to plants, making this fertile ground for expansion and development of DEB theory, particularly in application to plant SDMs (Schouten *et al.*, 2020). There are several reasons why DEB theory may be particularly useful for modelling resource allocation in plants. The fact that all plants require a very similar set of exogenous resources, namely light, water and nutrients, integrates well with the DEB modelling framework in which a small number of variables and fundamental processes linked through pathways represents an organism’s state as it changes through time (Ledder, 2014). Moreover, a number of features of terrestrial vascular plants, many of which arise because they have modular growth and are sessile, make resource allocation particularly fundamental to their life histories, and resolving the physiological basis of these resource allocation trade-offs represents one of the more thorny problems in plant biology that could be informed by a DEB approach. Moreover, while DEB processes are constrained through several core parameters, unlike optimality models, a DEB model does not make any explicit or implicit assumption about maximizing a particular outcome. Predictions from DEB models arise from processes operating from the ‘bottom-up’ rather than from ‘top-down’ constraints, which enhances their general applicability. There is thus much unrealized potential for using the DEB framework to address unanswered questions about how plants have navigated evolutionary trade-off landscapes to produce the diversity of life history strategies that we see on Earth and about how plants will navigate our rapidly changing world.

The goal of this article is to explore ways that the DEB modelling approach can be applied to terrestrial vascular plants, with a particular focus on resource allocation trade-offs. We focus on terrestrial vascular plants because most previous applications of DEB theory to photosynthetic organisms have been to microalgae and macroalgae (Lorena *et al.*, 2010; Muller, 2011; Lavaud *et al.*, 2020). Although trade-offs between allocation to reproduction and other functions (e.g, growth and survival) are important to understand, we focus on non-reproductive individuals in order to limit what would otherwise be a large scope for a single article. First, we discuss fundamental properties of terrestrial vascular plants, which for the sake of concision will hereafter be referred to as ‘plants’, emphasizing properties that distinguish them from unitary animals and that are relevant to a DEB theory for plants. Second, we translate these fundamental properties of plants into a list of stylized facts (*sensu*  Sousa *et al.*, 2010) that would be required for a generic DEB model of plants, as well as other stylized facts that would be useful for modelling plants in certain ecological scenarios, including responses to climate change. Finally, we present a generic DEB model for plants that builds on three published DEB models for plants (Kooijman, 2010; Ledder *et al.,* 2020; Schouten *et al.,* 2020).

## Leading a sessile, modular life while competing for similar resources

**Figure 1:**
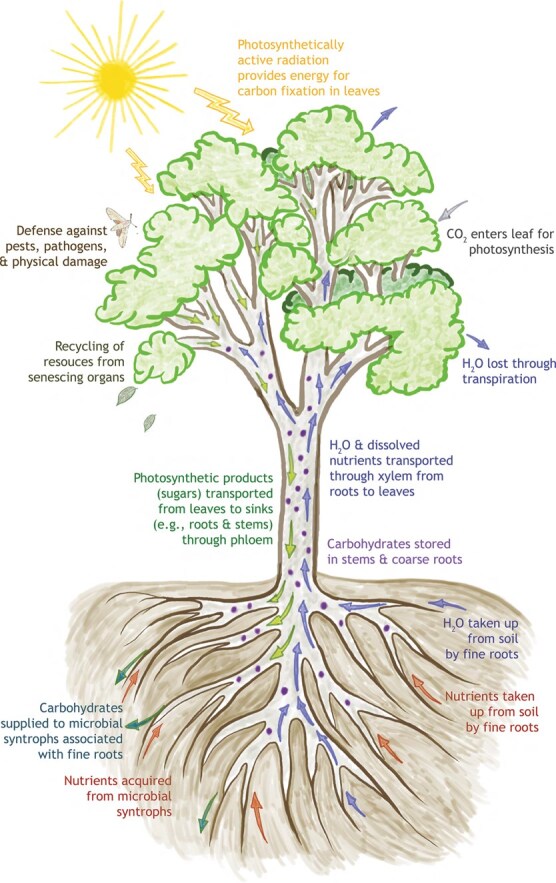
Depiction of key physiological and ecological processes affecting the growth and survival of plants described in Leading a sessile, modular life while competing for similar resources and corresponding to selected stylized facts described in Towards a Dynamic Energy Budget model for plants.

Many of the fascinating features of plants derive from a few fundamental properties (Fig. 1). First, plants have a modular body plan, which stands in contrast to animals, many of which are unitary organisms (key exceptions being some sessile animals, such as corals, bryozoans and sponges) (Hallé *et al.*, 1986; Tuomi and Vuorisalo, 1989; Oborny, 2019). Plants grow by reiteration of modules, which roughly correspond to the three vegetative organs: leaves, stems (including branches) and roots. Second, plants are sessile: they grow, reproduce and survive where they are rooted, which generates many and diverse consequences and results in fundamental differences from mobile animals (Karban *et al.*, 2016). Third, nearly all plants synthesize their own carbohydrates through photosynthesis and require a nearly identical set of exogenous resources to do this and to grow, namely the energy contained in sunlight, CO_2_, water and macronutrients like nitrogen (N), phosphorus (P) and potassium (K), as well as other micronutrients. Fourth, in plants, these exogenous resources are collected by different organs that operate in very different above and belowground environments. Leaves are the photosynthetic and light-capturing organs aboveground, and they require sufficient N, P, K and other nutrients as well as water, all of which are collected by roots, specifically narrow-diameter roots called fine roots. As the belowground portions of the plant are not photosynthetic, they must import photosynthetically fixed C from the leaves to grow and for maintenance respiration. The fact that different plant organs collect different resources in contrasting environments, combined with the modular nature of the organs, represents a key difference from most animals and unicellular organisms. In animals, the ability to adjust the body plan, cellular structures or physiology in order to acquire more of the most limiting resources is more constrained, and the organs of acquisition function in a more uniform environment, generally inside the body. Fifth, in the process of conducting photosynthesis, leaves lose water through evaporation in a process called transpiration, creating a nearly unavoidable photosynthesis-transpiration trade-off. Hence, acquiring one substrate (CO_2_) needed for assimilation of a resource (carbohydrates) by an organ (leaves) comes at the cost of loss of another resource (water) that is acquired by a different organ (fine roots). Moreover, water is very different from other resources. Plants require large volumes of water, but only a tiny fraction of water taken up by plant is directly involved in metabolism: most water is lost to the atmosphere through transpiration (Nobel, 2020). This, combined with modular growth and the water tran**s**port function of stems, creates a suite of complicated environment-dependent trade-offs in resource allocation to above and belowground plant parts. Managing these trade-offs is essential, as excessive water loss is detrimental, sometimes lethal, to the plant (Henry *et al.,* 2019; Wang *et al.*, 2020). Sixth, the life histories of plants vary among those that complete their life cycle in a single growing season (‘annuals’), two growing seasons (‘biennials’) or are longer lived (‘perennials’). Seventh, the life cycles of all plants are defined by alternation of generations, in which subsequent generations of individuals alternate between multicellular stages that are haploid and diploid. Our discussion of the application of DEB theory to plants will focus on the first five properties and will be biassed towards trees and perennial plants.

### Modularity, resource collection and growth in relation to phenotypic plasticity

For unitary organisms like most animals, ontogenetic development starts from a single cell and realizes a definite body plan, in which the number of organs and their sizes are strictly defined (Oborny, 2019). In contrast, in the modular development of plants, their ontogeny progresses by the re-iteration of finite developmental programs, each producing a module (Hallé *et al.,* 1986; Oborny, 2019). Modules are semi-autonomous, but structurally and functionally integrated, subunits that together comprise the whole organism (Lüttge, 2019). Modules in terrestrial vascular plants roughly correspond to units of organs, and there are three vegetative organs: roots, stems and leaves. The organization of cells and tissues within each organ is well defined, but with plasticity in structure and function. The architectural arrangement of the system of modules and their number, size and structure can also be flexible, similar to a construction toy (Hallé *et al.,* 1986; Tuomi and Vuorisalo, 1989; Oborny, 2019). Organs communicate with each other and share resources through a ramifying vascular system that connects all modules and that allows for the movement of water and nutrients in xylem tissue and of more complex molecules such as sugars, hormones and amino acids in phloem tissue (Lucas *et al.,* 2013). Units of each organ (e.g. individual leaves) are produced and senesced, often seasonally, and the integrated outcome of these processes over time comprises plant growth. Whether through active senescence, herbivory (the partial consumption of a plant by another organism) or other forms of physical damage, sometimes a large number of modules making up the plant body can become inactive or be lost altogether, but this rarely results in death of the plant, which is another key distinction from most unitary animals in their stress responses (Huey *et al.,* 2002).

The modularity of plants combined with their sessile nature potentially selects for phenotypic plasticity (the capacity of a genotype to produce different phenotypes depending on the environment; Via and Lande, 1985; Schlichting, 1986; Sultan, 1995; Arnold *et al.*, 2019), since plants cannot move to find better environments, as non-sessile animals can. This has a number of consequences for the resource allocation strategies of plants. Environmental conditions change through time due to seasonality and longer-term environmental changes, some of which are mediated through a plant’s own growth. In forests, for instance, there are vertical environmental gradients from forest floor to canopy, and as trees grow in height, they experience different environmental conditions through ontogeny (Yoda, 1974; Rijkers *et al.*, 2000; Bin *et al.,* 2022). Accordingly, there is corresponding variation in leaf and stem traits with tree height (Rijkers *et al.,* 2000; Couvreur *et al.,* 2018; Bin *et al.,* 2022).

Modularity and phenotypic plasticity are also key to feedbacks in which a plant’s own growth changes its environment and the resources available to it, for example, through self-shading (Donohue, 2003). Phenotypic plasticity can involve changes in resource allocation (Weiner, 2004; Weiner *et al.*, 2009) and occurs at all levels of biological organization: gene expression, molecules and organelles, cells, tissues, organs and phenology (Schlichting, 1986). Plant organs have distinct structures, stoichiometries and functions, but also have a dramatic degree of plasticity that is typically not present in the organs of unitary animals (Schlichting, 1986), but is key to understanding the responses of plants to environmental change and other stressors (Huey *et al.,* 2002; Arnold *et al.,* 2019). For example, because of the large amount of pigments and proteins that are involved in photosynthesis, leaves have high concentrations of nitrogen and phosphorus, and as a result their stoichiometries are defined by lower C:N and C:P ratios, compared with other plant organs. However, there is a large degree of interspecific and intraspecific variation in the structure and stoichiometry of leaves that depends not only on the environment the leaf and plant experience, but also on other factors (Lambers *et al.*, 1998). Many of the same environmental conditions that directly and indirectly affect photosynthetic function of a leaf (e.g. the intensity of light, atmospheric concentrations of CO_2_, leaf temperature) also affect the structure and stoichiometry of the leaf. These relationships between structure, function and the environment also depend on the species’ ecological strategy (Bin *et al.,* 2022). For instance, plant species that tend to grow quickly usually have thinner leaves with lower C:N ratios and faster rates of photosynthesis and respiration, compared with species of the same growth form (e.g. herbaceous plant, tree) that tend to grow more slowly (Lambers and Poorter, 1992; Poorter and Evans, 1998; Reich *et al.,* 1999; Reich, 2014). This interspecific coordination among traits and between traits and growth rates often corresponds to species’ distributions along soil resource and climate gradients (Cunningham *et al.*, 1999; Sterck *et al.,* 2011; Maire *et al.,* 2015; Wright and Westoby 2002; Weemstra *et al.* 2020). Likewise, plants of the same genotype or species that are grown in nutrient-poor soil will produce leaves that are smaller, thicker and tougher and have higher C:N and C:P ratios compared with plants grown in more fertile soil (Evans, 1989; Reich *et al.*, 1989; Aerts and Chapin, 2000; Liu *et al.*, 2012; Tatarko and Knops, 2018).

Leaves are displayed on stems, which include branches that consist of ramifying units. Stems are support organs, but also function in the transport and storage of photosynthetically fixed carbon in the form of sugars and starches (Hoch *et al.*, 2003), as well as of water and nutrients (Chapin *et al.*, 1990). The size and arrangement of stems dictates the overall structure of the plant aboveground (which is also true for roots belowground), but in many growth forms of plants the structure can be very plastic (Weiner, 2004; Ogawa, 2019). For example, among trees, individuals of the same species at the same age or size may have crowns of varying sizes and shapes resulting from modular, light-stimulated growth that produces branches and leaves in well-lit locations and senesces less productive leaves and the branches that display them in a way that is thought to maximize photosynthesis in a heterogeneously shaded environment (Field, 1983; Ackerly and Bazzaz, 1995; Niinemets and Kull, 1995).

Stems are connected to roots, which are generally underground and anchor the plant to the substrate, which is usually soil. Like stems aboveground, roots form a ramifying network belowground where they absorb water and nutrients, such as nitrogen, phosphorus, potassium and other mineral nutrients, from the soil and transport them to the aboveground portions of the plant. Roots also store many of the same endogenous resources that stems do (Wiley *et al.*, 2019). A distinction can be made between coarse (wider diameter) roots whose functions are mainly support/anchorage, storage and transport versus finer (narrower diameter) roots whose functions are mainly resource acquisition and transport (Waisel *et al.*, 2002; Weemstra *et al.,* 2016).

As a principal function of fine roots is to absorb resources from soil (McCormack *et al.,* 2015), they are the belowground analog of leaves, which absorb aboveground resources. Fine roots also are the principal sites for interactions with microorganisms in the soil, as described below. The structure of individual roots and the architecture of the root system as a whole are also very plastic (Hodge, 2004; Freschet *et al.,* 2021). Patterns of trait covariation involving durability and productivity trade-offs have been also found for roots, although they are often different from those for leaves (Comas and Eissenstat, 2004; Kochsiek *et al.*, 2013; Weemstra *et al.,* 2016; Weemstra *et al.,* 2020). Fine roots will proliferate into patches of soil that contain greater nutrient availability and will senesce in areas where nutrients have already been depleted (Robinson *et al.*, 1999; Hodge, 2004; Xia *et al.*, 2010), analogous to the production and senescence of leaves and branches to maximize light capture. In these respects, the modularity of plants allows them to function like optimal foragers (Charnov, 1976; Oborny, 1991; McNickle *et al.*, 2009). Moreover, the functioning of leaves and roots is complementary, in that each organ acquires types of resources that the other cannot and is influenced by different environmental conditions. This sets the stage for environment-dependent trade-offs in resource allocation above vs belowground that is a fundamental component of whole-organism phenotypic plasticity in plants (Poorter *et al.,* 2012).

### Defence

Plants cannot move to avoid risks of damage and death, which include risks caused by biotic interactions with predators, herbivores and other pests and pathogens (Grubb, 1992), but also by abiotic processes, such as falling branches and trees often caused by extreme weather events (Clark and Clark, 1991). As a result, there has been selection for self-defence that takes on many forms. Physical defence includes tough and robust tissues, like dense wood of trees that resists damage (Bultman and Southwell, 1976) and prickles, spines and thorns (Grubb, 1992). Plants are also some of the best chemists on Earth, and a single plant individual can produce thousands of different compounds, many of which are thought to function in defence (Fraenkel, 1959; Mithöfer and Boland, 2012). Chemical defensive molecules vary in stoichiometry and range from large, carbon-rich molecules, such as terpenoids, to smaller nitrogen-containing compounds, like alkaloids (Wink, 2010). Plants can also employ animals to defend them. For example, some plants, when damaged by herbivores, produce volatile compounds that attract predators of the herbivore to the site of damage, causing top-down regulation of herbivore populations (Tumlinson *et al.*, 1993; Paré and Tumlinson, 1999; Kessler and Baldwin, 2001). Intimate symbioses between plants and ants (known as myrmecophytic plants) have appeared many times in the evolutionary history of the flowering plants (Chomicki and Renner, 2015), with the ants aggressively defending their host plant against herbivores, and the plant provisioning the ants with resources, such as carbohydrate and fat-rich food and space in stems and specialized thorns for nesting (Davidson and McKey, 1993; Davies *et al.*, 2001). Of course, some animals, especially sessile ones, invest in defence against predation by producing chemical toxins, spines or other specialized defensive structures (Benard and Fordyce, 2003). However, it can be argued that that the principal modes of defence differ between mobile animals and plants, particularly as mobile animals invest heavily in behavioural strategies to avoid predation (Lima and Dill, 1990).

Of course, defences are costly and allocation of resources to functions reducing the risk of damage and death limit resources available to other functions, such as those promoting growth (Coley *et al.,* 1985; Simms and Rausher, 1987; Züst and Agrawal, 2017), which has been termed ‘the dilemma of plants: to grow or to defend’ (Herms and Mattson, 1992). As a result, the amount and type of defence covaries with other aspects of the life history of plant species, such as growth and survival rates (Coley *et al.,* 1985; Coley, 1987; Coley, 1988; Fine *et al.,* 2006; Agrawal, 2007; Imaji and Seiwa, 2010). As resource allocation to defence directly contributes to survival (Loehle, 1988; Loehle, 1996), but competes with physiological processes supporting other vital rates, such as growth and reproduction (Briggs and Schultz, 1990; Herms and Mattson, 1992; Weiner *et al.,* 2009), interspecific demographic trade-offs emerge that define ecological strategies of plants (Díaz *et al.,* 2016, Grime, 1977, Kobe and Coats, 1997, Reich, 2014, Rüger *et al.,* 2018, Russo *et al.,* 2021, Wright *et al.,* 2004).

### Storage and recycling

**Figure 2:**
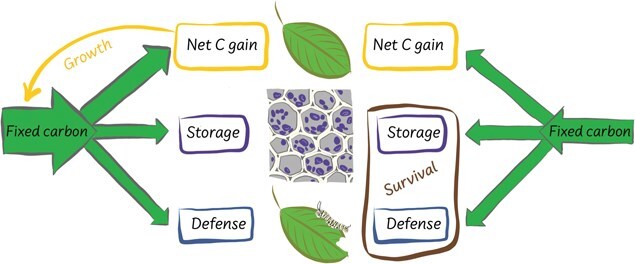
Trade-offs related to carbon allocation and their consequences for growth and survival. Left: Allocation of fixed carbon to functions supporting growth (e.g. photosynthesis, leaf production) may further increase the capacity to fix carbon, which may be an effective allocation strategy when resources are plentiful and the damage rate, such as from natural enemies or disturbances, is low. This strategy may also act to increase the total endogenous pool of fixed carbon (and other resources) available to allocate to alternative functions, including those promoting survival. Right: However, in environments where resources are more limited or highly variable in supply or where the damage rate is high, prioritization of allocation to growth may cause elevated mortality risk. In these environments, tissue construction costs are higher, implying that organs should be protected from damage, and survival may be increased by storing fixed carbon (e.g. as carbohydrates in stem and coarse roots) and allocating carbon to defence (e.g. construction of physically robust organs, synthesis of chemical defences), but this would come with a lost opportunity cost for growth. Resource allocation or functional trait expression may be plastically adjusted during a plant's lifetime (or may vary between individuals of the same species), depending on access to resources.

The sessile nature of plants combined with temporal variation in a plant’s environment should also select for the capacity to store, recycle and reallocate endogenous resources to different organs or tissues or locations within a plant’s body. Plants store essential resources, such as photosynthetically fixed carbon, water and nutrients (Chapin *et al.,* 1990; Hoch *et al.,* 2003). Plants, especially trees, store large amounts of water (Meinzer *et al.*, 2004; Köcher *et al.*, 2013) and carbohydrates (Plavcová and Jansen, 2015) in stem and root tissues. Stored water can be used to maintain hydraulic function (Meinzer *et al.,* 2004; Köcher *et al.,* 2013) and to flush new leaves, as adequate turgor pressure is required for growth (Chapotin *et al.*, 2006a). Carbohydrates are used as both materials and energy for the construction of new tissues and organs, as well as to pay the respiratory costs of maintaining existing structures. Allocation of fixed carbon to functions supporting growth can create a positive feedback that further increases the capacity to fix carbon, but this can come at the cost of increased risk of death if insufficient amounts of carbon are allocated to functions enhancing survival, such as defence and storage (Fig. 2). There is still debate as to whether the size of stored carbohydrate pools, which have consequences for survival during drought (Obrien *et al.*, 2014) and overwintering in temperate zones (Kobe, 1997; Canham *et al.*, 1999), is under active or passive control (Smith and Stitt, 2007; Sala *et al.*, 2012; Wiley and Helliker, 2012). While the sizes of the pools of stored resources in a plant are dynamic and a function of additions to and subtractions from the pools, active control of storage pools implies that a plant maintains a minimum amount of stored resources, whereas in passive control, additions and subtractions would be unregulated. This has important implications for understanding exactly how plants die, as discussed below in reference to DEB theory.

### Mutualistic relationships

The sessile nature of plants has also contributed to selection for the development of mutualisms with other eukaryotic organisms and bacteria. Most plants form a wide array of mutualistic relationships with animals, which include exchanges of resources for services requiring the ability to move (e.g. pollination, seed dispersal, defence) (Herrera and Pellmyr, 2002), as well as syntrophic relationships involving reciprocal exchange of resources (Cohn *et al.*, 1998; Brundrett, 2002; Brundrett and Tedersoo, 2018; Gupta and Sharma, 2021). Belowground syntrophic relationships involve plant roots and the bacteria and fungi that live in, on and near them and affect the acquisition of water and nutrients, which are patchily distributed in soil (Knops *et al.*, 2002; Schimel and Bennett, 2004; van der Putten *et al.,* 2013).

Plant roots can principally take up inorganic forms of nutrients, such as ammonium (NH_4_^+^), nitrate (NO_3_^−^) and orthophosphate (PO_4_^3−^) (Marschner, 1995). While some forms of nutrients that plants require are easily dissolved in the pore water of soil, and hence are more mobile through the soil matrix, other nutrients adhere more strongly to soil particles or may be occluded in them, making them harder for plant roots to access (Marschner, 1995; Tinker and Nye, 2000). Thus, less-mobile nutrients (*e.g.* phosphorus) form depletion zones around roots due to plant uptake (Giehl and von Wirén, 2014). In contrast to plants, microorganisms like bacteria and fungi produce an array of enzymes that can break the chemical bonds in the molecules that make up organic matter in soil. Plants generally must rely on decomposers to catabolize organic matter and convert the nutrients contained within it into plant-available forms (Knops *et al.,* 2002; Schimel and Bennett, 2004; van der Putten *et al.,* 2013). Thus, the challenges associated with acquiring nutrients from soil while being rooted in a single location combined with the costliness of root growth have likely selected for the evolution of plant-microbe syntrophies (Bergmann *et al.,* 2020; Tedersoo *et al.*, 2020).

Plant-microbe syntrophies are often obligate for the microorganism, but facultative for the plant. Well-known examples include bacteria of the family Rhizobiaceae possessing nitrogenase enzymes that fix dinitrogen within a plant organ specialized for housing the bacteria, the root nodule, which is characteristic of leguminous plants (Fabaceae) (Cohn *et al.,* 1998; Dos Santos *et al.*, 2012). Nearly 90% of all land plant species form symbioses with mycorrhizal fungi of several functional types in and on their roots (Smith and Read, 2008; Bonfante and Genre, 2010; Peay, 2016; Tedersoo and Brundrett, 2017). Mycorrhizal fungi form extensive mycelial networks in soil and in and on plant roots and produce hydrolytic enzymes lacking in plants that are capable of breaking down complex organic molecules, such as cellulose (Bonfante and Genre, 2010). Although there may be other benefits of association with microbes, such as defence (Bennett *et al.,* 2017; Tedersoo *et al.,* 2020), in syntrophic relationships, plants exchange photosynthetically fixed carbon for nutrients, principally N and P, supplied by the microbial symbiont. Plant roots take up water and nutrients unaided by microbiota, but there is a cost/benefit trade-off between this versus outsourcing these functions to symbionts (Bonfante and Genre, 2010; Bergmann *et al.,* 2020). The fact that up to 30% of recently produced photosynthates of a plant host can be allocated to mycorrhizal fungi (Nehls and Hampp, 2000; Hobbie, 2006; Soudzilovskaia *et al.,* 2015; Pringle, 2016; Thirkell *et al.*, 2020) suggests that this is a carbon cost that is worth paying. Not all symbionts are the same in terms of costs and benefits: some are more cooperative in that they supply more nutrients to plants, whereas others are more costly in terms of the carbon investment required (Kiers *et al.*, 2003; Kiers *et al.,* 2011). While these syntrophies are often termed mutualisms, they exist on a mutualism–parasitism continuum that is to some extent regulated by soil resource availability (Bronstein, 1994; Johnson *et al.*, 1997; Smith and Smith, 2013). Thus, the propensity of plants to form these resource-based syntrophies extends the consideration of trade-offs in resource allocation to encompass the competing interests of syntrophic partners.

### Phenotypic integration and trade-offs

There are many indirect consequences of resource allocation patterns, and some of these derive from the fact that plant organs have multiple functions and constraints, along with the necessity for phenotypic integration. Let us take the example of wood density, which is a commonly measured phenotypic trait of the stems of woody plants. Woody stems serve important support functions, allowing leaves to be displayed at tall heights and laterally along the tips of extending branches for light capture. Anatomically, wood is the secondary xylem of plant species that have secondary growth. Although xylem cells are dead at maturity, they serve essential hydraulic functions, operating like pipes to transport water and nutrients absorbed by fine roots from the soil to other parts of the plant (Carlquist, 1975). Lighter wood is often so because the xylem cells within it have larger-diameter lumens, which are also more efficient at conducting water (Hagen–Poiseuille law of laminar flow) (Tyree and Ewers, 1991). Water transport through xylem is critical for sustaining canopy photosynthesis (Tyree, 1999; Tyree, 2003; Couvreur *et al.,* 2018) and maintaining adequate turgor pressure for cell division and expansion during plant growth (Salisbury and Ross, 1992; Kozlowski and Pallardy, 1996; Kozlowski and Pallardy, 2002). Compared with narrower xylem cells, wider xylem cells can support faster water flow, but when plants become water limited, wider xylem are also more vulnerable to cavitation and air embolisms (Cruiziat *et al.*, 2002) that decrease the hydraulic conductivity of the xylem (Tyree and Sperry, 1989) and ultimately limit photosynthesis, producing trade-offs in xylem safety vs efficiency (Sperry *et al.*, 2008; Chave *et al.,* 2009; Gleason *et al.,* 2016). Wood density also influences water storage, or capacitance, in the stem, which can enhance survival during prolonged drought (Meinzer *et al.*, 2003; Chapotin *et al.*, 2006b; Ziemińska *et al.*, 2020), as well as resistance to pests, pathogens and physical damage (Chave *et al.,* 2009). By definition, denser wood has more mass per unit volume, and consequently more biomass must be invested to achieve a given amount of growth in volume for denser compared with lighter-wooded species (King *et al.*, 2006; Chave *et al.,* 2009 ; Russo *et al.,* 2010). Variation in wood density has also been linked to architectural and allometric differentiation among tree species, as lighter wood may be more efficient for vertical (height) growth, whereas denser wood may be more efficient for lateral growth (crown expansion and branching) (King *et al.*, 2005; van Gelder *et al.*, 2006; Anten and Schieving, 2010; Iida *et al.,* 2012).

It is clear that the way a single tissue, wood, is made produces resource allocation-based trade-offs related to productivity vs durability and safety vs efficiency, involving resistance to natural enemies and physical damage, tree allometry and architecture, hydraulic function and photosynthetic productivity, all of which have consequences for growth and survival rates (Chave *et al.,* 2009; Russo *et al.,* 2010). Analogous allocation-based trade-offs also operate for other tissues and organs and are thought to underlie trade-offs manifest at higher levels of biological organization (e.g. individuals, populations and species), such as between growth versus survival, growth versus reproduction and maturation versus growth (Salguero-Gómez, 2016; Rüger *et al.,* 2018; Detto *et al.,* 2021; Russo *et al.,* 2021).

### Modes of death and the importance of water

Exactly how organisms die is not well understood, even for humans, and it is especially not well understood for plants. By ‘plant death’ we mean, death of the entire organism (i.e*.* of all modules). Plants may die by several mechanisms, but some of these are particularly exacerbated by climate change: (1) a falling object (generally another plant), storms or extensive pest damage can cause a plant to experience an unsurvivable amount of catastrophic tissue loss; (2) a plant can be starved of carbohydrate resources and become unable to pay the cost of maintenance respiration (carbon starvation); and (3) a plant can experience catastrophic dysfunction of its hydraulic system and water balance that causes it to be unable to transport water to leaves or move stored resources from one location to the next (hydraulic failure).

Carbon starvation may occur if plants are unable to photosynthetically assimilate carbon for long periods of time, often as a result of water limitation, but possibly also due to extensive defoliation by pests or disturbances like hurricanes and to the accumulation of damage to photosynthetic organs and the tissues that supply them (Arellano *et al.*, 2019; Zuleta *et al.,* 2022). When limitations on photosynthesis are severe and prolonged, such as by drought, plants may die of carbon starvation when carbon fixation is limited and available pools of endogenous carbohydrates are insufficient to pay maintenance costs (McDowell *et al.,* 2008). The fundamental trade-off between CO_2_ fixation in photosynthesis and water loss via transpiration means that when plants experience water deficit the guard cells making up the stomatal pore close it, stopping CO_2_ from entering the leaf and ultimately stopping photosynthetic C-assimilation (Cowan, 1978b). Plants may then resort to stored carbohydrates to pay maintenance costs, grow or perform other necessary metabolic functions. However, these reserves may be depleted if photosynthesis is limited for a prolonged time period, and depletion of reserves is associated with increased mortality (Kobe, 1997; Sala *et al.*, 2010; Obrien *et al.,* 2014). Because large plants like trees can store large amounts of carbohydrates in stems and roots, it is likely that small perennial or juvenile plants with limited reserves are much more vulnerable to death by carbon starvation.

Conversely, large plants like trees may be more vulnerable to death by hydraulic failure, due to the difficulty of raising water to leaves at tall heights. Although carbon starvation may result from water limitations, hydraulic failure is a distinctly different process. Under severe water limitation, xylem cavitations that impede water flow may be so extensive that the plant may pass a threshold beyond which xylem can no longer be refilled with water, causing plants to die by hydraulic failure (Sperry and Tyree, 1988; Hacke and Sauter, 1996; McDowell *et al.,* 2008). This may happen even when a plant still has ample stored carbohydrates (McDowell *et al.,* 2008) because the functioning of xylem and phloem, which transports sugars and other metabolites, are linked in that sufficiently low plant water potential also impedes the plant’s ability to access and transport reserves (Plavcová and Jansen, 2015). To use an economic analogy, dying from ‘carbon starvation’ is analogous to running out of money in the bank and being unable to buy food, whereas dying from ‘hydraulic failure’ is analogous to having money in the bank, but being unable to access the bank to withdraw it. Water balance and hydraulic processes in the plant therefore place fundamental constraints on resource assimilation and translocation, and so while water is a resource, like light and nutrients, it plays a fundamentally different role.

## Application of DEB models to terrestrial vascular plants

An attractive feature of DEB theory is that, rather than elaborating the functions of different morphological components of an organism, as we have done in the previous section for plants, it abstracts an organism’s functioning in terms of material fluxes of substrates in processes of assimilation, growth and turnover (Jusup *et al.,* 2017), while accounting for feedbacks, and so, in principle, it can capture complex somatic growth dynamics, as well as trade-offs and scaling relationships that affect rates of growth, survival and reproduction (Sousa *et al.,* 2010; Ledder, 2014; Jusup *et al.,* 2017). If starting *ab initio*, it would be straightforward to write an overview of animal physiology, with a level of detail analogous to that in the preceding section, which would highlight a large list of ‘key’ properties for inclusion in a generic DEB model for an animal. The breakthrough achieved with Kooijman’s DEB theory (Kooijman, 1986; Kooijman, 2001) is that it evolved to a unified framework with a generic ‘standard’ animal model involving a small number of state variables, which operates as a template from which many variants have been derived (e.g. Kooijman, 2010; Jusup *et al.,* 2017). The most important simplifications for the standard model (all potentially relaxed in variants) are as follows: (i) subdivision of living tissue into just two types, structure that requires maintenance and reserve that does not; (ii) assuming idealized types of homeostasis; (iii) restriction to a single resource input (‘food’) with the effect of the other fundamental resource (dioxygen absorbed via different organs) subsumed into model parameters (yield coefficients); and (iv) neglect of phenotypic plasticity. Some ‘key’ properties, notably interspecific co-variation of parameters, are emergent properties of the standard DEB model, not assumptions.

For a number of reasons discussed below, plants pose a much greater challenge for DEB theory compared with unitary animals. While simple submodels can be used to connect an animal’s rate of resource collection to its structural masses, the models that connect carbon capture to leaf mass and water and nutrient uptake to root mass are more complicated and should potentially incorporate hydraulic processes and the costs and effects of water transport. Indeed, there are many detailed models of organ-specific physiological processes in plants, including water transport through xylem in stems (e.g. Tyree and Sperry, 1988; Sperry *et al.,* 2012; Wolf *et al.*, 2016; Couvreur *et al.,* 2018), uptake of water from soils by roots (e.g. Couvreur *et al.*, 2012; Couvreur *et al.*, 2014), nutrient uptake by roots (e.g. Leitner *et al.,* 2010), photosynthesis (e.g. Farquhar *et al.*, 1980; Evans *et al.*, 1993; von Caemmerer, 2000) and stomatal conductance (e.g. Way *et al.*, 2011; Wang *et al.*, 2020). Plant models that integrate environment-dependent organ-specific physiological processes, alternative resource allocation strategies operating at the whole-plant level, are crucial because they can better capture the demographic consequences of complex trade-offs. Because of the properties of plants described above, as well as others not addressed here, plant growth, survival and reproduction are subject to these trade-offs in ways that are arguably more complicated than in unitary organisms, making it harder to derive a single ‘standard’ DEB model for plants analogous to the one for animals, as we elaborate below.

Below is a list of properties that we think should be considered when formulating a DEB model that predicts somatic growth of a non-reproductive plant that interacts with its environment. Including all of them would lead to an excessively complex model and for this reason we advocate selecting a subset to serve as the broad generalizations (stylized facts) for a generic model analogous to the standard DEB model for animals. In the next section, we outline one such generic model. For particular applications, the selection and prioritization of stylized facts will depend on the desired level of generality and on the focal biological questions. However, a key requirement is that the plant’s performance not only changes as a result of the environmental conditions, but that the plant can plastically adjust aspects of its form and function to changing conditions in ways that allow a more adaptive response (i.e. beneficial for fitness) to its current environment. Since the environment changes in time at various scales, and plants change their own environment through their growth, this modelling capacity is arguably necessary if we are to build models that can predict plant responses to climate change, including changes in species’ habitat and geographic distributions (Nicotra *et al.,* 2010; Nicotra and Davidson, 2010; Valladares *et al.,* 2014).

The first nine properties are candidate stylized facts that can support generic DEB models for plants. In the text below, italicized words refer to named components of existing DEB theory, whereas words in quotes refer to components that would need to be added to or significantly modified in existing DEB theory. Properties range from those that are already relatively easily accommodated by existing DEB methodology to those that are more challenging but do not require fundamentally new additions to DEB theory to those that require new fundamental thinking and additions to existing DEB theory in order to be accommodated.

P1. **There are four environmental *resources*—CO**_**2**_**, photosynthetically active radiation, water and nutrients.** All nutrients (e.g. N, P, K) can be treated together with the most limiting nutrient being the nutrient resource currency used in the model (Kooijman, 2001; Ledder *et al.,* 2020). DEB models for plants that consider only two of these resources explicitly have been developed and analysed (e.g. Ledder *et al.,* 2020), but such models cannot account for all of the resources that commonly limit plant growth. Carbon dioxide and nutrients are clearly resources, as they are substrates for biosynthetic reactions. While atmospheric CO_2_ itself is not limiting, because of the photosynthesis–transpiration trade-off, the amount of CO_2_ inside the leaf at the sites of carboxylation can be limiting. Photosynthetically active radiation (solar radiation in the range of photosynthetically active wavelengths, commonly known as light) cannot be stored, but is required to energize the carbon fixation reactions. Moreover, competition for light is thought to have been responsible for the evolution of molecules and organs (stems) that allow plants to grow tall and shade their neighbours. Hence, we consider light a *resource*. Very little of the water that a plant uses is chemically incorporated into metabolites. However, water in soil can be depleted by and stored in the plant, and when water was incorporated into a DEB model for plants as an environmental control on shoot growth, via its effects on photosynthesis, but not as a depletable resource, this created unrealistic root growth dynamics during drought (Schouten *et al.,* 2020). Thus, we suggest water should be considered a *resource*, but as elaborated in more detail below, it functions differently as a *resource*, compared with CO_2_, light or nutrients. A more complex DEB model for plants might also include submodels that describe the drivers of the availability of these resources with respect to the environment (P2) and plant-environment feedbacks (P7).

P2. **Key properties of the *environment* are air relative humidity (RH) and temperature**. The distinction we make between resources and environmental properties is that resources are essential substrates for metabolic processes, whereas environmental properties are those that, in combination with other factors, modulate the rates of resource acquisition and metabolic processes and thereby also influence the heat, water, nutrient and carbon balances of the plant (Gates, 1980; Kearney *et al.*, 2021). We consider the environment to be that in the immediate spatial vicinity of the plant (that is, the microenvironment), regardless of the timescale under consideration. We distinguish air temperature and RH, which are easily measured by widely used environmental sensors, as more fundamental climatic variables, from which other important climatic variables, such as vapour pressure deficit (VPD), can be calculated. Many DEB applications adopt a minimally complex treatment of temperature, assuming all rates to have identical or near-identical temperature dependence, which would clearly be incompatible with what is known about the complex effects of temperature on plants (Wahid *et al.*, 2007; Slot *et al.*, 2019; Smith *et al.*, 2019). For example, the temperature of the leaf influences the rates of CO_2_ fixation (Farquhar *et al.,* 1980; Yamori *et al.*, 2014; Dusenge *et al.*, 2019) and cellular respiration (Heskel *et al.,* 2016; Dusenge *et al.,* 2019) and hence growth (Way and Oren, 2010). Leaf temperature is determined not only by air temperature, but also by the size and shape of the leaf and the amount of direct versus indirect solar radiation heating the leaf, among other factors (Leigh *et al.*, 2017). Temperature also affects the rate of respiration (Atkin *et al.*, 2005; Lambers and Ribas-Carbo, 2005; Slot *et al.,* 2019; Smith *et al.,* 2019). Air temperature interacts with RH to cause variation in the leaf-to-air VPD, which, combined with the water potential in the leaf, is the driving force for transpiration that affects not only leaf temperature, but also stomatal functioning, which together affect the rate of carbon fixation (Farquhar *et al.*, 2001; Way *et al.,* 2011) and hydraulic function (Cowan, 1978a; Henry *et al.,* 2019). In a more complex DEB model for plants, the physical properties of the soil could be considered, along with rainfall, as a key environmental property, as both influence the rate of belowground resource supply (P1).

P3. **There should be two different *structures* for collecting the resources** (Kooijman, 2001). Leaves collect CO_2_, intercept light and synthesize sugars, and water and nutrients are collected by fine roots. Each *structure* has its own stoichiometry.

P4. **For larger or woody plants, a DEB model should explicitly account for the energetic roles of living tissue in stems and coarse roots.** These roles include transporting and storing fixed C, water and nutrients between the *structures* (leaves and fine roots) and in supporting and displaying the *structures*. Accounting for the functions of stems and coarse roots is important, as they figure prominently in models of succession and competition for light, even for shorter stature vegetation (Detto *et al.,* 2021). Moreover, empirical studies of biomass allocation have argued against lumping stems and leaves into shoots, and instead advocated for considering mass fractions of leaves, stems and roots separately (Poorter *et al.,* 2012). It has been proposed that the xylem in stems and coarse roots could be considered *product*, in that it is dead and does not directly require maintenance costs (Kooijman, 2001; Schouten *et al.,* 2020), although it still serves vital transport functions that provides essential support functions affecting resource collection (e.g. carbon fixation and nutrient uptake) and remobilization. If the definition of a product can be modified so that its properties (e.g. size and transport capacity) can impact plant functioning, then xylem can be considered *product*. Alternatively, DEB theory needs a new definition for a metabolism and maintenance-free *structure* (which we refer to as ‘structure’) that can affect the functioning of resource collection by leaves and fine root *structures* and also be able to mediate death.

P5. **The multifaceted role of water must be accounted for.** A generic DEB model for plants should include photosynthesis–transpiration and safety–efficiency trade-offs and allow for these trade-offs to influence plant growth and survival. Moreover, a generic plant DEB model should allow for nutrients and water to independently limit plant growth, even though both are collected by the fine roots.

P6. **Resource-specific *reserves* and dynamics.** There should be resource-specific *reserves* and dynamics that differ depending on their utilization by different *structures* and the ‘structure’ or product representing stems (P4). For example, when a plant makes new leaves, it must mobilize both carbohydrates and nitrogen from *reserves* in the appropriate stoichiometric ratio to construct a leaf, as constrained by that particular species and its environment. The stoichiometric ratio for roots will be different because the nature of the tissues is different, but also because all of the carbon required to build new roots must come from *reserve*, whereas that is not true of the leaves (and vice versa for nitrogen). Likewise, the dynamics of the same reserve, such as carbohydrates, in different *structures*, such as leaves versus stems, are different. Generally, leaves have faster dynamics, as they export carbohydrates at the end of the day for longer-term storage in stems. Carbohydrates in stems are remobilized less frequently, for example, to repair damage or break bud in the spring. Accurately capturing these sorts of dynamics is essential for a model to capture responses to stress and seasonal growth patterns.

P7. **Plant-environment feedbacks.** The microenvironment that a plant experiences is not only defined by resources (P1) and environmental variables (P2), but also by how those are affected by the properties and functioning of the plant itself, that is, plant-environment feedbacks. The capacity for feedbacks in which the plant impacts the state of its own local environment is essential. Plant-environment feedbacks include self-shading caused by the plant over-topping itself as it grows (e.g. Ackerly, 1999). In addition, soil water and nutrient availability are not only influenced by rainfall and the physical properties of the soil, but also, critically, can be depleted around roots by plant uptake, which is a driving force for root growth (Lambers *et al.,* 1998; Couvreur *et al.,* 2012; Farrior *et al.,* 2013; Sulis *et al.,* 2019). The capacity for plant-environment feedbacks to arise can be integrated into a plant DEB model with appropriate physiological, biophysical and soil hydraulics process models describing relationships across the soil–plant–atmosphere continuum. Since plant-environment feedbacks change across the lifetime as the plant grows, a DEB model for plants would ideally capture these feedbacks across the full life cycle of the plant (Kearney, 2019). Although animals can change their own environment as ecosystem engineers, they usually do so in a way from which they benefit, which is not true of resource depletion in plants. Mobile animals can also deplete resources, but they can move to a new environment to find more resources, whereas a plant (and sessile animals) can only grow beyond its own depletion zone or wait for resources to be replenished.

P8. **Growth capacities should be modular.** This can be accomplished by allowing for the semi-autonomous production and senescence or loss of the *structures*.

**Figure 3:**
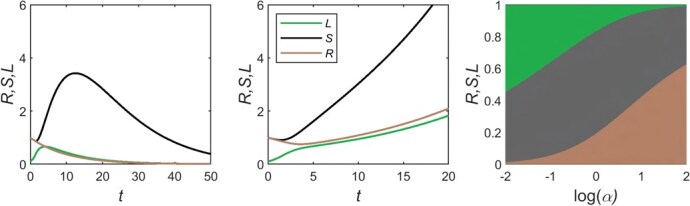
Simulation results for a model based on the Ledder *et al.* (2020) root-shoot model that used passive local rules for transport of resources and growth of roots and shoots (stems plus leaves), but extended to a setting in which the stem is considered as a separate *structure* with its own SU. Two scenarios for the root-stem-leaf model were considered for a starting condition in which the mass of leaves is initially deficient: a perfectly efficient SU determined by the Liebig’s minimum rule in which growth is dictated by the single scarcest resource (a) and the PCSU that is based on more realistic biochemical assumptions and has a third lower efficiency than the minimum rule SU (**b**, **c**). In (a) and (b), the x-axis is time (t) and the y-axis is the mass of each organ. With the minimum rule (a), leaves (L, green) initially grow, but ultimately the masses of both leaves and roots (R, brown) go to zero as the plant becomes completely dominated by stems (S, black). With the PCSU, leaves, stems and roots, maintain growth and mass > 0. In (c), the x-axis is the log of α (the ratio of carbon availability to nitrogen availability) and the y-axis is the mass of each organ, shown as a proportion of the total plant mass. For each value of α the vertical variation in shading shows the relative sizes of the root (brown), stem (black) and leaf (green) masses. When carbon is more plentiful (right-most side of the x-axis), the plant has a greater proportion of roots, whereas when nitrogen is easier to collect (left-most side of the x-axis), then the plant has a greater proportion of leaves. For all values of α, the size of the stem is determined by the extent to which the stem SU limits resource availability to the root and shoot SUs and not on any intrinsic value of stems. These SUs are described in Ledder *et al.* (2020), and further details are provided in Supplementary Appendices 1 and 2.

**P9. Mechanisms for balanced growth among organs.** Resource allocation to roots, stems and leaves is a fundamental feature of how plant phenotypes are modified depending on the environment, and a mechanism for achieving realistic mass ratios between these organs during plant growth is essential to a generic DEB model for plants. The modularity of DEB theory in principle allows the linkage of several *structures* and their *synthesizing units (SUs)* comprising different parts of an organism, as has been done in models of plant growth accounting for allocation to roots versus shoots in terrestrial plants (Kooijman, 2001; Ledder *et al.,* 2020; Schouten *et al.,* 2020). A ‘sharing the surplus’ mechanism has been shown to yield optimal growth for a large variety of scenarios in a model consisting of roots and shoots (Ledder *et al.,* 2020); however, this mechanism is not adequate for a model that interposes stems as an additional SU between the roots and the leaves. With the assumption that SUs are perfectly efficient with regard to the utilization of the limiting resource (the minimum rule SU in Ledder *et al.,* 2020, there is never a scenario in which the stems are required to share both a photosynthate surplus and a nutrient surplus. Hence, the system evolves to a state in which the plant fails because the leaves and roots are unable to grow (Fig. 3a). With the alternative assumption of a biochemical SU model in which there is some inefficieny in the SUs (the parallel complementary SU in Ledder *et al.,* 2020), the leaves and roots are able to grow (Fig. 3b), but the extra resource control owing to the stem’s central position produces stem growth that would be out of proportion to the overall value of the stem to the plant for most species and that is not adaptive with respect to the need for stems (Fig. 3c) (see Supplementary Appendix 1, with mathematical details in Supplementary Appendix 2). Thus, while the mechanism of sharing the surplus in a model with roots, stems and leaves in series can maintain the growth of all organs, our work suggests that this mechanism would need to be supplemented by restrictions that tie stem growth to stem value. The generic DEB model for plants that we present below (with details in Supplementary Appendix 3) provides a framework for one possible way to solve this issue.

Characterizing the fate of a plant in real environments will require adding to any growth model modules describing response to stress, including mechanisms causing death. The following modules would allow the model plant to respond to common and important environmental stressors:

M1. **Defence.** There should be a way to account for trade-offs between durability vs productivity and safety vs efficiency of the *structures*, along with their resulting feedbacks on growth and survival. This would involve a mechanism for preventing biomass loss (e.g. chemical or physical defences or immune response) that increases survival, but reduces resources available for growth and reproduction and also accounts for lost opportunity costs of not having invested those resources in growth (Fig. 2). Defence could be treated like *maturity*. That is, there could be a variable representing the cumulative investment in defence, and some parameters like tissue turnover could depend on that variable. Defence may also be considered a *product* that reduces the background mortality rate in relation to a damage parameter, similar to the way longevity is handled in DEB theory, coupled with a form of a κ parameter to capture the trade-offs with growth and reproduction (Muller and Nisbet, 2000). Different types of defences would have different stoichiometries. Defence likely requires some maintenance costs (e.g*.* turnover of enzymes to produce chemical defences).

M2. **Recycling of resources.** Resorption of resources from senescing modules to the *reserves* should be allowed and may depend on exogenous environmental conditions (e.g. light or soil nutrient and water availability), as well as endogenous conditions, namely water balance.

M3. **Plant death and lifespan.** As described above, how plants die is not well understood, but three inter-related mechanisms involve carbon starvation, hydraulic failure and accumulation of damage to tissues and organs supporting resource acquisition and metabolism. In DEB theory, one of the ways that organisms die is because their energy balance falls below that required for maintenance, and the analogue of this in plants is carbon starvation. The extent to which plants are resistant or tolerant of sources of damage, thereby decreasing their hazard rate (death probability per unit time), determines their lifespan, and there is substantial interspecific variation in the survival rates of plant species (Condit *et al.*, 1995; Russo *et al.,* 2021), often linked to the amount of investment in defence (Loehle, 1988; King *et al.,* 2006; Imaji and Seiwa, 2010). There is scope for a unified and mechanistic theory of plant death in DEB theory, as well as in other modelling approaches.

Finally, we list some plant properties that may require either modification of some core concepts of current DEB theory or considerable elaborations with consequential complexity.

E1. **Environment-dependent plasticity.** There should be environment-dependent plasticity in how *structure* is built and in resource allocation between *structures*, the ‘structure’, *products* and *maturity/reproduction*, both during ontogeny (e.g. as trees grow up through the canopy, their microenvironment changes) and for modules of *structure* in different parts of the plant that may experience different microenvironments (*e.g.* sun vs shade leaves). The incorporation of plasticity requires careful consideration because thermodynamic constraints and the strong homeostasis assumption in DEB theory requires that variation in the composition of organisms only occurs via variation in the proportion of *reserves* vs *structure*, not by stoichiometric variation in *structure* itself (Kooijman, 2001; Sousa *et al.,* 2010). We note that this issue is not specific to DEB models of plants. There is scope for new general theory that better connects DEB principles with existing ecological theory on plasticity, and progress is being made in this area (Récapet *et al.*, 2019; Koch and De Schamphelaere, 2020; Mounier *et al.,* 2020).

E2. **Functioning of reserves.** The way *reserves* function may need modification in application to a generic DEB plant model. In Kooijman’s DEB theory, reserves function both like a ‘pass through account’ that temporarily holds assimilated resources prior to being metabolized and as a store for future use—including meeting metabolic requirements under stress conditions (Kooijman, 2001). As described above, plants store carbohydrates, water and nutrients to be used later and have specialized structures for doing so (e.g. amyloplasts and vacuoles). Storage of resources increases survival during resource-limiting periods, but also trades-off against growth and, presumably, reproduction. To accommodate this, the *reserves* associated with each *structure* could be partitioned into compartments that have different temporal dynamics. This issue is not specific to plant models; see Martin *et al.* (2017) for an animal example. Especially with longer-lived plants, processes influencing the storage and remobilization of resources will occur at very different time scales. Ledder *et al.* (2020) demonstrated potential complexity in the dynamics with even the simplest plant caricature where model analysis requires rather careful consideration of time scale separation, which has been elaborated by Pfab *et al.* (in revision).

E3. **Plant–microbe interactions.** Some would argue that given the ubiquity and importance of plant-microbe interactions in nature, syntrophic relationships should also be included in a generic DEB model for plants. However, plants in principle can survive on their own in the absence of these syntrophies, and so these are not included as minimally necessary to describe the functioning of a plant. That said, the coupling of DEB models representing syntrophic partners has been applied to the syntrophic relationship defining coral (Muller *et al.*, 2009), as well as nodulation in soybean (Klanjšček *et al.*, 2017) and discussed in light of trade-offs in root:shoot allocation (Ledder *et al.,* 2020). While we do not elaborate on the possibilities here, we view the modularity of DEB theory as particularly amenable to modelling plant–microbe syntrophies.

## Towards a DEB model for plants

It might appear surprising that there is no ‘standard’ DEB model for plants 35 years after the formulation of the standard animal DEB model (Kooijman, 1986), the ancestor of a diverse lineage of DEB models. Like plants, animals require inputs from the environment that are processed by different organs (food and water via the gut, oxygen from the lungs). However, the key to the simplicity of the standard model is that it is possible to specify a set of stylized facts supporting a representation that gives primacy to one of the inputs, energy. Oxygen is, by default, assumed to be always available as needed, with special cases such as hypoxia (Lavaud *et al.,* 2019). This narrow focus is possible because of an animal’s tightly integrated physiological homeostasis across multiple levels of suborganismal organization. However, as recognized by Kooijman (2001), the much weaker regulation of the organs in a plant implies that that the dynamics of interactions among plant organs likely resemble interactions among species in ecological models. From a DEB perspective, this implies that each of root, stem and shoot has at least one structural mass. Homeostasis between reserve(s) and structure *within* organs of plants have thus been modelled utilizing well-established DEB concepts (*e.g.*  Kooijman, 2010), but apparent whole-plant homeostasis is an *emergent property* from the dynamics of interacting organs.

The requirements in points P1–P9 in the preceding section represent a candidate list of stylized facts for a generic DEB model of a plant, at least for modelling somatic growth (it does not describe reproduction). Table 1 summarizes the extent to which these and modules M1–M3 are incorporated into three previously published models, each written with different objectives. The parameter-rich, complex model in Kooijman’s (2010) book aimed to demonstrate rigorous implementation of DEB principles into a plant model. Ledder *et al.* (2020) aimed to elucidate the ecological and evolutionary dynamics resulting from the assumption that organs share the surplus elemental matter they cannot use. Schouten *et al.* (2020) aimed to integrate DEB and microclimate models to describe plant species distributions. With such contrasting aims, it is reasonable that each chose to base model assumptions on different stylized facts. We have no immediate, well-defined route from these stylized facts that could inform a generic, tractable DEB model for terrestrial vascular plants, but now propose a starting point with a new model that treats water in an analogous matter to the standard animal model’s treatment of oxygen. It is inspired by Kooijman's (2010, Chapter 5) plant model, but differs from the Kooijman model in fundamental aspects as outlined below and in Table 1—namely as it has fewer state variables and many fewer parameters. In addition, it reduces the number of distinct reserve variables and treats translocation differently.

**Table 1:** Summary of the extent to which our proposed eight candidate stylized facts (P1–P8) and three growth model modules (M1–M3) are incorporated, or can easily be incorporated, in existing DEB models for plants

	Candidate stylized fact	Kooijman (2010, Chapter 5)	Ledder *et al.* (2020)	Schouten *et al.* (2020)	This study
P1	Four environmental *resources*: CO_2_, light, water, nutrients	Yes	CO_2_, light, and nutrients implicitly	Yes, all explicitly	CO_2_, nutrients
P2	Air RH and temperature	Temperature straightforward, no RH	No	Yes, explicitly with microclimate submodule NicheMapR (Kearney and Porter, 2017)	Temperature straightforward, no RH
P3	Two different *structures* for collecting the resources with different stoichiometry	Yes	Yes	Yes	Yes
P4	Energetic roles of living tissue in stems and coarse roots	No, stems and coarse roots modelled as metabolic products	No (but see P9, Fig. 3 and Supp. Appendices 1 and 2 for a modification of the Ledder et al. model in which stems and coarse roots are metabolic *structures*, but lack physiological function)	No	No, stems and coarse roots modelled as metabolic products
P5	Multifaceted role of water	No	No	Partial (water potential can affect photosynthesis, but water shortage did not stimulate root growth)	No
P6	Resource-specific *reserves* and dynamics	Three reserves per structure, no resource-specific reserve dynamics	No	No	Two reserves per structure, no resource-specific reserve dynamics
P7	Plant-environment feedbacks	No	No	No	No
P8	Modular growth	Yes	Yes	Yes	Yes
P9	Mechanisms for balanced growth among organs	Sharing the surplus; passive translocation	Sharing the surplus	Passive lossless or dissipative translocation	Sharing the surplus
M1	Defence	No	No	No	No
M2	Recycling of resources	Yes	Yes	Yes	Yes
M3	Plant death	No	No	No	No

Light refers to photosynthetically active radiation.

As already noted, resource acquisition rates are highly dependent on physical conditions, such as water potential. However, the model presented here only describes potential resource acquisition rates based on the capacity of the biochemical machinery of a plant; additional modules are needed to specify the impact of environmental conditions on the uptake of resources. Like Kooijman (2020), Ledder *et al.* (2020) and Schouten *et al.* (2020), it describes the dynamics of energy (carbon) and nitrogen (standing in for all resources acquired by fine roots) sources and sinks and organ growth and development with a minimum number of state variables and parameters and with minimum use of active control mechanisms for resource allocation. It achieves simplification from Kooijman’s model in two ways: (i) the interacting organs (root and shoot) only share resource that is surplus to their own requirements and (ii) we reduce the number of reserve compartments within each structure from three to two. Model notation, details of derivation and model equations are in Supplementary Appendix 3.

**Figure 4:**
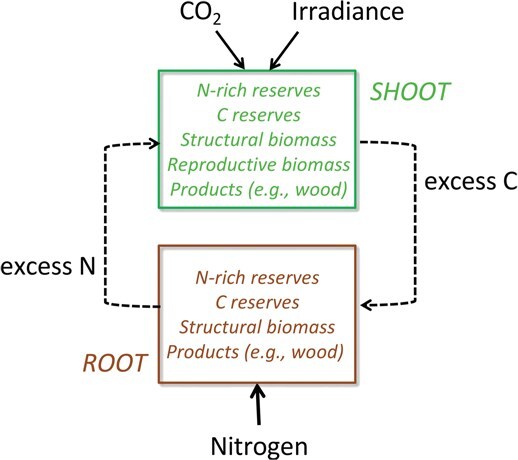
Conceptual model overview showing the relationship and composition of the two functional units, roots and shoots. The boxes list the types of biomass distinguished in the DEB model, all of which are state variables, with the exception of ‘products’. For more detail, see Fig. 5.

The model assumes that a plant consists of two mutually dependent functional units with distinct roles in the acquisition of resources from the environment. These units are called ‘shoots’ and ‘roots’ (Fig. 4). The shoot acquires all energy and carbon from the environment, whereas the root takes up all other nutrients (lumped as a single variable). In principle, these units could be spatially integrated, arranged in proximity or organized further apart with a transport system connecting them (stems), as in most terrestrial plants. This begs the question whether the transport system should be modelled as a separate unit. However, our provisional explorations in this direction indicate this would require additional constraints on the allocation of resources to leaf, root and transport systems. Here, we consider the transport system as integrated within the root and shoot, as Ledder *et al.* (2020) showed that local control via surplus sharing is fully adequate to account for plant growth, and in many situations is optimal. This choice may need to be revisited for applications where explicit representation of physiological functions of stem is important.

Shape considerations are important in DEB theory, as surface area-to-volume ratios affect rates of metabolism and resource acquisition. Metabolism in vascular plants is organized in structures having the approximate shape of warped sheets (such as, among other structures, leaves and the cambium and phloem in dicotyledon trees) and tubes (such as, among other structures, stems in herbaceous plants and root hairs). These two shapes share an important characteristic: their surface area-to-volume ratio is approximately constant, provided that sheets do not grow in thickness and that tubes are cylinders that grow only in length. In organisms with a constant functional surface-to-volume or structural biomass ratio (so-called V1-morphs in DEB modelling; Kooijman, 2010), resource acquisition and allocation rates scale similarly with size. This feature greatly aids in constraining model complexity and also implies that growth is indeterminate in a constant environment. Biologically, this approach allows us to separate the interactions responsible for whole-plant homeostasis from those that regulate the final size of the plant (e.g. light limitation and the requirement that an increasing percentage of resources is invested in structures supporting the leaves and fine roots). These can easily be added to the model in particular applications if so desired. However, a fundamental difference between our approach and Kooijman’s approach remains. Whereas Kooijman’s model retains a tight coupling between resource acquisition and reserve utilization rates (as both groups of rates similarly depend on plant size and shape), that coupling is more complex if spatially explicit descriptions of self-limiting features, such as self-shading, were to be included. The model’s current formalism does allow for self-limitation provided the effect can be described using its state variables. In this, it parallels the simpler model of Ledder *et al.* (2020). A tight coupling presupposes a high level of physiological integration of functions at the individual level, which may be appropriate when modelling the energy budgets of an animal (i.e. the paradigm in DEB modelling), but is less defensible when modelling those of plants, in which the parts operate at a much higher level of autonomy.

In line with DEB theory, our proposed generic DEB model for plants distinguishes four types of biomass based on metabolic function.

1. ***Structural biomass*** performs the necessary metabolic functions to remain viable. To maintain functional performance, a continuous supply of maintenance energy is needed, e.g. to replace damaged biomolecules and restore ion gradients over membranes.
2. ***Reserve biomass*** can be metabolized to maintain the functional performance of structural biomass and may include metabolically active biomass that may be sacrificed without loss of vitality. We consider two types of reserves in both shoots and roots: reduced carbon and nitrogen-rich reserves, which consist of organic compounds, such as proteins with a metabolic function (e.g. Rubisco) (Fig. 3). Plants also store nitrate in vacuoles, but the fraction of inorganic nitrogen to total nitrogen is usually very low, especially in non-crop species. Therefore, we ignore nitrate reserves and note that the model may not be adequately describing the performance of crop species in highly fertilized soils.
3. ***Reproductive biomass*** consists of gametes and all of the biomass of reproductive organs, including that required for fertilization and dispersal of offspring (not explicitly modelled here).
4. ***Products*** include any kind of biomass or secondary metabolite that is neither subject to maintenance nor turnover inside the plant. Products include compounds that provide physical support (e.g. cellulose in cell walls and lignin in wood) and those that are involved in functions like protection, but may ultimately leave the plant, such as waxes at the interface between environment and epidermis and volatiles like terpenoids.

**Figure 5:**
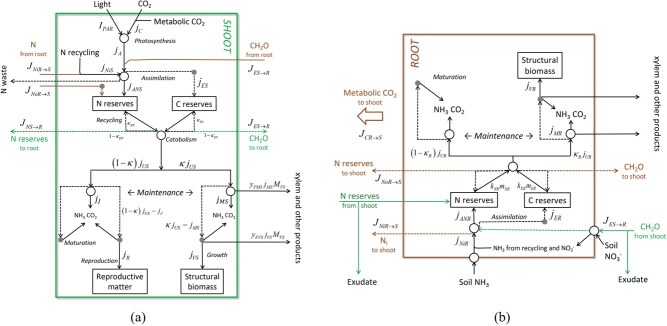
Schematic of mass and energy fluxes in (**a**) shoot (S) and (**b**) root (R) according to the DEB model for plants derived in Supplementary Appendix 2. The model distinguishes two types of reserve in shoot and root: nitrogen-rich and carbon reserve; both types consist of organic compounds. State variables are boxed. SUs are represented by circles (open circles are SUs that may reject part of arriving substrates; closed circles are SUs that process all available substrates). Broken lines represent rejection fluxes. Note that in both shoot and root assimilation, the formation of nitrogen-rich reserve (‘N Reserves’), which may contain carbon, takes priority over that of reduced carbon reserve (‘C Reserves’). Hence, the SU producing the latter receives rejected reduced carbon (‘CH_2_O’) from the former. Notation mostly follows DEB convention (Kooijman, 2010) and is fully specified in Supplementary Table 1 in Supplementary Appendix 2. Absolute fluxes are represented by capital *J*, while lowercase *j* denote structural biomass-specific fluxes; arrows in subscripts refer to fluxes that are translocated from root to shoot or vice versa (note that both shoot and root contribute to ‘Metabolic CO_2_’ in (a)).

The model relies heavily on the concept of the *SU*, which is a stylized representation of the biochemical machinery that performs the energy and material transformations in DEB models. SUs are particularly useful for describing transformation rates involving multiple reactants or substrates (Kooijman, 2010, Chapter 3). We need *SUs* (shown in Figs 4 and 5) describing single and double substrate processes. For the latter we use *SUs* that simultaneously process two substrates in parallel. In both cases, the capacities of *SUs* specifying the growth rates of *structure* are constrained by resource input rates (determined by the amount of *structure* and the resource availability in the environment, as well as the rejection flux from the partner *SU*). Once resources are acquired, *SUs* specifying the rates of internal processes are unconstrained, so that all available substrates (single-substrate SUs) or all substrates in short supply (the stoichiometrically limiting substrate in double-substrate SUs) will be fully processed.

In standard DEB models of animals, a fixed-fraction $\kappa$ of the catabolic flux is spent on somatic maintenance and growth, while the remainder is used to support maturation and reproduction functions. In contrast to metabolically highly integrated animals, plants show a high level of plasticity with regards to size at sexual maturity, and the high level of autonomy of organs allows them to plastically respond to environmental variation. Therefore, it seems likely that $\kappa$strongly varies with environmental conditions and possibly depends on the state of maturity and/or reproductive buffer (Muller *et al.,* 2019). We ignore those complications here. For further details the reader is referred to Supplementary Appendix 3.

## Discussion: future priorities for DEB modelling of plants

Environmental changes facing plants include changing climatic regimes, particularly temperature and rainfall, as well as increased exposure to and damage from pests and pathogens (Roy *et al.,* 2014). In order to predict the responses of plants to such changes, the appropriate dependence of metabolic processes on abiotic and biotic environmental conditions must be included in models. Below we outline areas where development of new modelling approaches is required to do so in DEB theory.

The clear top priority for future work focusing on climate change is to integrate models, like ours in the preceding section, to mechanistic treatments of water in plants. The only DEB model to date that has attempted this is Schouten *et al.* (2020), which used a modified version of the Farquhar model of photosynthesis (Farquhar *et al.,* 2001). Schouten *et al.*’s model demonstrated adaptive growth in response to environmental drivers, including water, in some circumstances. Water limitation reduced shoot growth, but contrary to expectation, it did not stimulate root growth, which could only be stimulated in the model by nutrient limitation. Thus, growth in water-limited scenarios was not accurately predicted qualitatively. This happened because water uptake by the plant did not feedback to reduce the soil moisture in a fully integrated soil–plant–atmosphere continuum in the model. Instead, soil water potential was treated as a fixed environmental condition. However, such plant-environment feedbacks are essential for realistic models of plant growth and survival at both the individual and population levels (Ehrenfeld *et al.*, 2005). Our description of plant processes above points to seven key roles of water to consider including in DEB model for plants in which water may be limiting: (i) regulating the rate of C fixation by the leaves through the effects of xylem water potential and air RH on stomatal aperture (photosynthesis–transpiration trade-off); (ii) depletion of soil water caused by the uptake of water by plants; (iii) storage of water in leaves, stems and roots; (iv) regulating whether growth can occur, given sufficient C and nutrients, through the effects of turgor pressure on cell expansion; (v) enabling transport of dissolved nutrients from roots to leaves; (vi) enabling the mobilization of C reserves through phloem function; and (vii) mediating death through hydraulic failure, a point discussed further below. Given that greater atmospheric CO_2_ concentrations are causing increases in the water-use efficiency of plants, with consequences for the productivity and diversity of vegetation, as well as for carbon, nutrient and hydrological cycles (Guerrieri *et al.,* 2019; Baca Cabrera *et al.*, 2021), it is essential for plant DEB models to accurately capture the interactions between photosynthesis and transpiration in relation to plant-environment feedbacks.

Another pressing need is a more mechanistic treatment of plant death (death of the entire organism). In DEB theory for animals, an organism can die in two ways, through the metabolic and physical consequences of accumulated damage from oxidative stress, environmental toxins or natural enemies that increase the hazard rate (Jager *et al.*, 2011; Civitello *et al.*, 2018) and through carbon starvation, defined as the inability to pay for maintenance costs (Kooijman, 2010). In the latter mechanism, many DEB applications include an *ad hoc* treatment of death by starvation, e.g. by hypothesizing an inverse correlation between hazard rate and reserve density. There are clear analogies here with what we know about how plants die, but modifying existing DEB theory to accommodate these mechanisms is necessary.

We end by noting two ways in which progress in developing DEB models for plants could impact DEB theory targeting other taxa. First, there is the issue of adaptive and acclimatory phenotypic variation*.* DEB theory is well equipped to handle adaptation over timescales comparable with or longer than individual lifetimes (e.g. Troost *et al.*, 2005). Acclimation, or plasticity in phenotypes at the individual level, where it is considered, is often treated in *ad hoc* ways (Zonneveld and Kooijman, 1989). In plants, individual-level plasticity in phenotypic expression can be considered a form of habitat selection (Donohue, 2003) and allows plants to respond to microenvironmental variation in space and time, which is fundamental to modelling their responses to climate change at multiple scales (De Frenne *et al.,* 2013; Harwood *et al.,* 2014). At a coarse scale, environment-driven variation in biomass allocation to leaves, stems and roots (i.e. growth of these organs) can be considered individual-level plasticity, as it can fundamentally change the form and function of the plant (Poorter *et al.,* 2012). At least for biomass allocation to roots versus shoots, several models, including DEB models, can account for this level of plasticity (*e.g.*  Sterck *et al.,* 2011; Ledder *et al.,* 2020; Schouten *et al.,* 2020). However, these coarser-scale allocation patterns are influenced by environmental-driven variation in phenotypic expression at lower levels of biological organization, such as at the organ or tissue level. Given that all of these forms of plasticity are likely to influence species’ responses to environmental change and resulting shifts in distribution and diversity (*e.g.*  Valladares *et al.,* 2014), a fundamental challenge for DEB theorists is to develop formalism for phenotypic plasticity at multiple levels of biological organization.

A second connection with broader DEB theory involves interspecific covariation of parameters. Some of the most compelling evidence of the value of Kooijman’s standard model is that with some additional, plausible assumptions, it predicts interspecific covariation of parameters (Lika *et al.*, 2011). The predictions on interspecific variation are testable (e.g. Maino *et al.*, 2014) and support the ‘add-my-pet’ (AmP) project (Marques *et al.,* 2018) that to date has enabled DEB parameter estimation for, at the time of writing, well over 3000 species, almost all animals. Notwithstanding plants’ capacity for phenotypic plasticity, interspecific covariation among plant traits (Reich, 2014) and among demographic rates (Salguero-Gómez *et al.,* 2016; Russo *et al.,* 2021) is well documented. Any future generic DEB model for plants will achieve applicability to a much wider range of plants if the supporting theory leads to robust predictions of interspecific connections in parameter values. Lastly, a generic DEB model for plants would facilitate models of plant–animal interactions, such as plant–herbivore interactions, as has been done for corals (Muller *et al.,* 2009) and of how plants create and modify habitats for animals through effects such as on vegetation structure, shading and soil moisture.

## Conclusions

A number of features of plants, many of which arise because they are modular and sessile, make trade-offs related to resource allocation particularly fundamental to their life histories. Predicting how the distribution of diversity will shift with climate and other environmental changes is of urgent conservation importance but relies heavily on accurate and comprehensive models that capture the physiological basis of these trade-offs and their consequences for demography. This is particularly true of plants because, unlike mobile animals, plants cannot easily move to find suitable environments. These models are currently lacking, and while significant development of DEB theory may be required, there is much unrealized potential for using the DEB framework to address unanswered questions about how plants have navigated evolutionary trade-off landscapes and to predict the responses of plants to environmental change.

## Author Contributions

S.E.R. wrote the sections focusing on plant biology, derived from a keynote address at the Seventh Annual International Symposium and Thematic School on Dynamic Energy Budget Models, and in consultation with R.M.N., G.L. and E.M.; and developed the list of plant properties, modules and extensions important for a generic DEB model for plants. G.L. contributed the model and results for the three-SU root–stem–leaf model. E.B.M. developed the generic DEB model for plants. All authors contributed to the writing of the paper.

## Data Availability

No data were used in this study.

## Funding

This work was conducted as a part of the (A Dynamic Energy Budget Model for Trees) Working Group at the National Institute for Mathematical and Biological Synthesis, supported by the National Science Foundation through NSF Award #DBI-1300426, with additional support from The University of Tennessee, Knoxville. R.M.N. had support from National Science Foundation grant EF-1921356. Any opinions, findings and conclusions or recommendations expressed in this material are those of the author(s) and do not necessarily reflect the views of the National Science Foundation.

## Conflict of Interest

The authors declare no conflicts of interest.

## Supplementary Material

Russo_et_al_2022_ConsPhys_SupplApp1-2_coac061

## References

[ref1] Ackerly D (1999) Self-shading, carbon gain and leaf dynamics: a test of alternative optimality models. Oecologia 119: 300–310. 28307752 10.1007/s004420050790

[ref2] Ackerly DD, Bazzaz FA (1995) Leaf dynamics, self-shading and carbon gain in seedlings of a tropical pioneer tree. Oecologia 101: 289–298. 28307049 10.1007/BF00328814

[ref3] Aerts R, Chapin FS (2000) The mineral nutrition of wild plants revisited: a re-evaluation of processes and patterns. Advances in Ecological Research 30: 1–67.

[ref4] Agrawal AA (2007) Macroevolution of plant defense strategies. Trends Ecol Evol 22: 103–109. 17097760 10.1016/j.tree.2006.10.012

[ref5] Anten N, Schieving F (2010) The role of wood mass density and mechanical constraints in the economy of tree architecture. Am Nat 175: 250–260. 20028240 10.1086/649581

[ref6] Arellano G, Medina NG, Tan S, Mohamad M, Davies SJ (2019) Crown damage and the mortality of tropical trees. New Phytol 221: 169–179. 30067290 10.1111/nph.15381

[ref7] Arnold PA, Kruuk LEB, Nicotra AB (2019) How to analyse plant phenotypic plasticity in response to a changing climate. New Phytol 222: 1235–1241. 30632169 10.1111/nph.15656

[ref8] Atkin OK, Bruhn D, Hurry VM, Tjoelker MG (2005) The hot and the cold: unravelling the variable response of plant respiration to temperature. Funct Plant Biol 32: 87–105. 32689114 10.1071/FP03176

[ref9] Baca Cabrera JC, Hirl RT, Schäufele R, Macdonald A, Schnyder H (2021) Stomatal conductance limited the co2 response of grassland in the last century. BMC Biol 19: 50. 33757496 10.1186/s12915-021-00988-4 PMC7989024

[ref10] Benard MF, Fordyce JA (2003) Are induced defenses costly? Consequences of predator-induced defenses in western toads, bufo boreas. Ecology 84: 68–78.

[ref11] Bennett JA, Maherali H, Reinhart KO, Lekberg Y, Hart MM, Klironomos J (2017) Plant-soil feedbacks and mycorrhizal type influence temperate forest population dynamics. Science 355: 181. 28082590 10.1126/science.aai8212

[ref12] Bergmann J, Weigelt A, van der Plas F, Laughlin DC, Kuyper TW, Guerrero-Ramirez N, Valverde-Barrantes OJ, Bruelheide H, Freschet GT, Iversen CM et al. (2020) The fungal collaboration gradient dominates the root economics space in plants. Sci Adv 6: eaba3756. 32937432 10.1126/sciadv.aba3756 PMC7458448

[ref13] Bin Y, Li Y, Russo SE, Cao H, Ni Y, Ye W, Lian J (2022) Leaf trait expression varies with tree size and ecological strategy in a subtropical forest. Funct Ecol 36: 1010–1022.

[ref14] Bloom AJ, Chapin FS, Mooney HA (1985) Resource limitation in plants-an economic analogy. Annual Review of Ecology and Systematics 16: 363–392.

[ref15] Bonfante P, Genre A (2010) Mechanisms underlying beneficial plant–fungus interactions in mycorrhizal symbiosis. Nat Commun 1: 48. 20975705 10.1038/ncomms1046

[ref16] Briggs MA, Schultz JC (1990) Chemical defense production in lotus corniculatus l. II. Trade-offs among growth, reproduction and defense. Oecologia 83: 32–37. 28313239 10.1007/BF00324630

[ref17] Briscoe NJ, Elith J, Salguero-Gómez R, Lahoz-Monfort JJ, Camac JS, Giljohann KM, Holden MH, Hradsky BA, Kearney MR, McMahon SM et al. (2019) Forecasting species range dynamics with process-explicit models: matching methods to applications. Ecol Lett 22: 1940–1956. 31359571 10.1111/ele.13348

[ref18] Bronstein JL (1994) Our current understanding of mutualism. Q Rev Biol 69: 31–51.

[ref19] Brundrett MC (2002) Coevolution of roots and mycorrhizas of land plants. New Phytol 154: 275–304. 33873429 10.1046/j.1469-8137.2002.00397.x

[ref20] Brundrett MC, Tedersoo L (2018) Evolutionary history of mycorrhizal symbioses and global host plant diversity. New Phytol 220: 1108–1115. 29355963 10.1111/nph.14976

[ref21] Bultman JD, Southwell CR (1976) Natural resistance of tropical American woods to terrestrial wood-destroying organisms. Biotropica 8: 71–95.

[ref22] Burrows MT, Schoeman DS, Richardson AJ, Molinos JG, Hoffmann A, Buckley LB, Moore PJ, Brown CJ, Bruno JF, Duarte CM et al. (2014) Geographical limits to species-range shifts are suggested by climate velocity. Nature 507: 492–495. 24509712 10.1038/nature12976

[ref23] von Caemmerer S (2000) Biochemical Models of Leaf Photosynthesis. CSIRO Publishing, Collingwood, Australia

[ref24] Canham C-D, Kobe R-K, Latty E-F, Chazdon R-L (1999) Interspecific and intraspecific variation in tree seedling survival: effects of allocation to roots versus carbohydrate reserves. Oecologia 121: 1–11. 28307877 10.1007/s004420050900

[ref25] Carlquist S (1975) Ecological Strategies of Xylem Evolution. University of California Press, Berkeley, CA, USA

[ref26] Chapin FSI, Shulze ED, Mooney HA (1990) The ecology and economics of storage in plants. Annu Rev Ecol Syst 21: 423–447.

[ref27] Chapotin SM, Razanameharizaka JH, Holbrook NM (2006a) Baobab trees (adansonia) in Madagascar use stored water to flush new leaves but not to support stomatal opening before the rainy season. New Phytol 169: 549–559. 16411957 10.1111/j.1469-8137.2005.01618.x

[ref28] Chapotin SM, Razanameharizaka JH, Holbrook NM (2006b) A biomechanical perspective on the role of large stem volume and high water content in baobab trees (Adansonia spp.; bombacaceae). Am J Bot 93: 1251–1264. 21642189 10.3732/ajb.93.9.1251

[ref29] Charnov EL (1976) Optimal foraging: the marginal value theorem. Theor Popul Biol 9: 129–136. 1273796 10.1016/0040-5809(76)90040-x

[ref30] Chave J, Coomes D, Jansen S, Lewis SL, Swenson NG, Zanne AE (2009) Towards a worldwide wood economics spectrum. Ecol Lett 12: 351–366. 19243406 10.1111/j.1461-0248.2009.01285.x

[ref31] Chomicki G, Renner SS (2015) Phylogenetics and molecular clocks reveal the repeated evolution of ant-plants after the late miocene in Africa and the early miocene in Australasia and the Neotropics. New Phytol 207: 411–424. 25616013 10.1111/nph.13271

[ref32] Civitello DJ, Fatima H, Johnson LR, Nisbet RM, Rohr JR (2018) Bioenergetic theory predicts infection dynamics of human schistosomes in intermediate host snails across ecological gradients. Ecol Lett 21: 692–701. 29527787 10.1111/ele.12937

[ref33] Clark DB, Clark DA (1991) The impact of physical damage on canopy tree regeneration in tropical rain forest. J Ecol 79: 447–457.

[ref34] Clausen J, Nobs MA, Björkman O, Keck DD, Hiesey WM (1940) Experimental Studies on the Nature of Species. Carnegie Institution of Washington, Washington, D.C.

[ref35] Cohn J, Bradley Day R, Stacey G (1998) Legume nodule organogenesis. Trends Plant Sci 3: 105–110.

[ref36] Coley PD (1987) Interspecific variation in plant anti-herbivore properties: the role of habitat quality and rate of disturbance. New Phytol 106: 251–263.

[ref37] Coley PD (1988) Effects of plant growth rate and leaf lifetime on the amount and type of anti-herbivore defense. Oecologia 74: 531–536. 28311759 10.1007/BF00380050

[ref38] Coley PD, Bryant JP, Chapin FS (1985) Resource availability and plant antiherbivore defense. Science 230: 895–899. 17739203 10.1126/science.230.4728.895

[ref39] Comas LH, Eissenstat DM (2004) Linking fine root traits to maximum potential growth rate among 11 mature temperate tree species. Funct Ecol 18: 388–397.

[ref40] Condit R, Hubbell SP, Foster RB (1995) Mortality rates of 205 Neotropical tree and shrub species and the impact of a severe drought. Ecol Monogr 65: 419–439.

[ref41] Cooke SJ, Sack L, Franklin CE, Farrell AP, Beardall J, Wikelski M, Chown SL (2013) What is conservation physiology? Perspectives on an increasingly integrated and essential science†. Conserv Phys Ther 1: cot001. 10.1093/conphys/cot001 PMC4732437 27293585

[ref42] Couvreur V, Ledder G, Manzoni S, Way DA, Muller EB, Russo SE (2018) Water transport through tall trees: a vertically explicit, analytical model of xylem hydraulic conductance in stems. Plant Cell Environ 41: 1821–1839. 29739034 10.1111/pce.13322

[ref43] Couvreur V, Vanderborght J, Beff L, Javaux M (2014) Horizontal soil water potential heterogeneity: simplifying approaches for crop water dynamics models. Hydrol Earth Syst Sci 18: 1723–1743.

[ref44] Couvreur V, Vanderborght J, Javaux M (2012) A simple three-dimensional macroscopic root water uptake model based on the hydraulic architecture approach. Hydrol Earth Syst Sci 16: 2957–2971.

[ref45] Cowan IR (1978a) Stomatal behaviour and environment. In RD Preston, HW Woolhouse, eds, Advances in Botanical Research, Vol. 4. Academic Press, pp. 117–228

[ref46] Cowan IR (1978b) Stomatal behaviour and environment. Adv Bot Res 4: 117–228.

[ref47] Cruiziat P, Cochard H, Ameglio T (2002) Hydraulic architecture of trees: main concepts and results. Ann For Sci 59: 723–752.

[ref48] Cunningham SA, Summerhayes B, Westoby M (1999) Evolutionary divergences in leaf structure and chemistry, comparing rainfall and soil nutrient gradients. Ecol Monogr 69: 569–588.

[ref49] Davidson DW, McKey D (1993) Ant-plant symbioses: stalking the chuyachaqui. Trends Ecol Evol 8: 326–332. 21236183 10.1016/0169-5347(93)90240-P

[ref50] Davies SJ, Lum SKY, Chan R, Wang LK (2001) Evolution of myrmecophytism in western Malesian *Macaranga* (euphorbiaceae). Evolution 55: 1542–1559. 11580014 10.1111/j.0014-3820.2001.tb00674.x

[ref51] De Frenne P, Rodríguez-Sánchez F, Coomes DA, Baeten L, Verstraeten G, Vellend M, Bernhardt-Römermann M, Brown CD, Brunet J, Cornelis J et al. (2013) Microclimate moderates plant responses to macroclimate warming. Proc Natl Acad Sci 110: 18561. 24167287 10.1073/pnas.1311190110 PMC3832027

[ref52] Detto M, Levine JM, Pacala SW (2021) Maintenance of high diversity in mechanistic forest dynamics models of competition for light. Ecol Monogr 92: e1500.

[ref53] Díaz S, Kattge J, Cornelissen JHC, Wright IJ, Lavorel S, Dray S, Reu B, Kleyer M, Wirth C, Colin Prentice I et al. (2016) The global spectrum of plant form and function. Nature 529: 167–171. 26700811 10.1038/nature16489

[ref54] Donohue K (2003) Setting the stage: phenotypic plasticity as habitat selection. Int J Plant Sci 164: S79–S92.

[ref55] Dos Santos PC, Fang Z, Mason SW, Setubal JC, Dixon R (2012) Distribution of nitrogen fixation and nitrogenase-like sequences amongst microbial genomes. BMC Genomics 13: 162. 22554235 10.1186/1471-2164-13-162 PMC3464626

[ref56] Dusenge ME, Duarte AG, Way DA (2019) Plant carbon metabolism and climate change: elevated co2 and temperature impacts on photosynthesis, photorespiration and respiration. New Phytol 221: 32–49. 29983005 10.1111/nph.15283

[ref57] Ehrenfeld JG, Ravit B, Elgersma K (2005) Feedback in the plant-soil system. Annu Rev Env Resour 30: 75–115.

[ref58] Evans JR (1989) Photosynthesis and nitrogen relationships in leaves of c₃ plants. Oecologia 78: 9–19. 28311896 10.1007/BF00377192

[ref59] Evans JR, Jakobsen I, Ögren E (1993) Photosynthetic light-response curves: 2. Gradients of light absorption and photosynthetic capacity. Planta 189: 191–200.

[ref60] Farquhar GD, von Caemmerer S, Berry JA (1980) A biochemical model of photosynthetic co2 assimilation in leaves of c3 species. Planta 149: 78–90. 24306196 10.1007/BF00386231

[ref61] Farquhar GD, von Caemmerer S, Berry JA (2001) Models of photosynthesis. Plant Physiol 125: 42–45. 11154292 10.1104/pp.125.1.42 PMC1539321

[ref62] Farrior CE, Dybzinski R, Simon AL, Pacala SW (2013) Competition for water and light in closed-canopy forests: a tractable model of carbon allocation with implications for carbon sinks. Am Nat 181: 314–330. 23448882 10.1086/669153

[ref63] Field C (1983) Allocating leaf nitrogen for the maximization of carbon gain: leaf age as a control on the allocation program. Oecologia 56: 341–347. 28310214 10.1007/BF00379710

[ref64] Fine PVA, Miller ZJ, Mesones I, Irazuzta S, Appel HM, Stevens MHH, Saksjarvi I, Schultz JC, Coley PD (2006) The growth-defense trade-off and habitat specialization by plants in Amazonian forests. Ecology 87: 150–162. 10.1890/0012-9658(2006)87[150:tgtahs]2.0.co;2 16922310

[ref65] Fisher RA, Koven CD, Anderegg WRL, Christoffersen BO, Dietze MC, Farrior CE, Holm JA, Hurtt GC, Knox RG, Lawrence PJ et al. (2018) Vegetation demographics in earth system models: a review of progress and priorities. Glob Chang Biol 24: 35–54. 28921829 10.1111/gcb.13910

[ref66] Fraenkel GS (1959) The raison d'etre of secondary plant substances. Science 129: 1466. 13658975 10.1126/science.129.3361.1466

[ref67] Freschet GT, Roumet C, Comas LH, Weemstra M, Bengough AG, Rewald B, Bardgett RD, De Deyn GB, Johnson D, Klimešová J et al. (2021) Root traits as drivers of plant and ecosystem functioning: current understanding, pitfalls and future research needs. New Phytol 232: 1123–1158. 33159479 10.1111/nph.17072

[ref68] Gates DM (1980) Biophysical Ecology. Springer, New York, NY

[ref69] van Gelder HA, Poorter L, Sterck FJ (2006) Wood mechanics, allometry, and life-history variation in a tropical rain forest tree community. New Phytol 171: 367–378. 16866943 10.1111/j.1469-8137.2006.01757.x

[ref70] Giehl RFH, von Wirén N (2014) Root nutrient foraging. Plant Physiol 166: 509–517. 25082891 10.1104/pp.114.245225 PMC4213083

[ref71] Gleason SM, Westoby M, Jansen S, Choat B, Hacke UG, Pratt RB, Bhaskar R, Brodribb TJ, Bucci SJ, Cao K-F et al. (2016) Weak tradeoff between xylem safety and xylem-specific hydraulic efficiency across the world's woody plant species. New Phytol 209: 123–136. 26378984 10.1111/nph.13646

[ref72] Grime JP (1977) Evidence for the existence of three primary strategies in plants and its relevance to ecological and evolutionary biology. Am Nat 111: 1169–1194.

[ref73] Grubb PJ (1992) A positive distrust in simplicity—lessons from plant defences and from competition among plants and among animals. J Ecol 80: 585–610.

[ref74] Guerrieri R, Belmecheri S, Ollinger Scott V, Asbjornsen H, Jennings K, Xiao J, Stocker Benjamin D, Martin M, Hollinger David Y, Bracho-Garrillo R et al. (2019) Disentangling the role of photosynthesis and stomatal conductance on rising forest water-use efficiency. Proc Natl Acad Sci 116: 16909–16914. 31383758 10.1073/pnas.1905912116 PMC6708355

[ref75] Gupta VSRV, Sharma A (2021) Rhizosphere Biology: Interactions Between Microbes and Plants. Springer, Singapore, p. 356

[ref76] Hacke U, Sauter JJ (1996) Xylem dysfunction during winter and recovery of hydraulic conductivity in diffuse-porous and ring-porous trees. Oecologia 105: 435–439. 28307135 10.1007/BF00330005

[ref77] Hallé F, Harper JL, Rosen BR, White J (1986) Modular growth in seed plants. Philos Trans R Soc London B, Biol Sci 313: 77–87.

[ref78] Harwood TD, Mokany K, Paini DR (2014) Microclimate is integral to the modeling of plant responses to macroclimate. Proc Natl Acad Sci 111: E1164. 24569867 10.1073/pnas.1400069111 PMC3977301

[ref79] Henry C, John GP, Pan R, Bartlett MK, Fletcher LR, Scoffoni C, Sack L (2019) A stomatal safety-efficiency trade-off constrains responses to leaf dehydration. Nat Commun 10: 3398. 31363097 10.1038/s41467-019-11006-1 PMC6667445

[ref80] Herms DA, Mattson WJ (1992) The dilemma of plants: to grow or defend. Q Rev Biol 67: 283–335.

[ref81] Herrera CM, Pellmyr O (2002) Plant–Animal Interactions: An Evolutionary Approach. Blackwell Science, Ltd., Oxford, UK, p. 328

[ref82] Heskel MA, O’Sullivan OS, Reich PB, Tjoelker MG, Weerasinghe LK, Penillard A, Egerton JJG, Creek D, Bloomfield KJ, Xiang J et al. (2016) Convergence in the temperature response of leaf respiration across biomes and plant functional types. Proc Natl Acad Sci 113: 3832. 27001849 10.1073/pnas.1520282113 PMC4833281

[ref83] Hobbie EA (2006) Carbon allocation to ectomycorrhizal fungi correlates with belowground allocation in culture studies. Ecology 87: 563–569. 16602286 10.1890/05-0755

[ref84] Hoch G, Richter A, Körner C (2003) Non-structural carbon compounds in temperate forest trees. Plant Cell Environ 26: 1067–1081.

[ref85] Hodge A (2004) The plastic plant: root responses to heterogeneous supplies of nutrients. New Phytol 162: 9–24.

[ref86] Huey RB, Carlson M, Crozier L, Frazier M, Hamilton H, Harley C, Hoang A, Kingsolver JG (2002) Plants versus animals: do they deal with stress in different ways? Integr Comp Biol 42: 415–423. 21708736 10.1093/icb/42.3.415

[ref87] Iida Y, Poorter L, Sterck FJ, Kassim AR, Kubo T, Potts MD, Kohyama TS (2012) Wood density explains architectural differentiation across 145 co-occurring tropical tree species. Funct Ecol 26: 274–282. 10.1890/11-2173.1 24669729

[ref88] Imaji A, Seiwa K (2010) Carbon allocation to defense, storage, and growth in seedlings of two temperate broad-leaved tree species. Oecologia 162: 273–281. 19763628 10.1007/s00442-009-1453-3

[ref89] Iwasa Y, Roughgarden J (1984) Shoot/root balance of plants: optimal growth of a system with many vegetative organs. Theor Popul Biol 25: 78–105.

[ref90] Jager T, Albert C, Preuss TG, Ashauer R (2011) General unified threshold model of survival—a toxicokinetic-toxicodynamic framework for ecotoxicology. Environ Sci Technol 45: 2529–2540. 21366215 10.1021/es103092a

[ref91] Johnson NC, Graham JH, Smith FA (1997) Functioning of mycorrhizal associations along the mutualism–parasitism continuum*. New Phytol 135: 575–585.

[ref92] Jusup M, Sousa T, Domingos T, Labinac V, Marn N, Wang Z, Klanjšček T (2017) Physics of metabolic organization. Phys Life Rev 20: 1–39. 27720138 10.1016/j.plrev.2016.09.001

[ref93] Karban R, Orrock JL, Preisser EL, Sih A (2016) A comparison of plants and animals in their responses to risk of consumption. Curr Opin Plant Biol 32: 1–8. 27262943 10.1016/j.pbi.2016.05.002

[ref94] Kearney M, Porter W (2009) Mechanistic niche modelling: combining physiological and spatial data to predict species’ ranges. Ecol Lett 12: 334–350. 19292794 10.1111/j.1461-0248.2008.01277.x

[ref95] Kearney M, Porter WP (2004) Mapping the fundamental niche: physiology, climate, and the distribution of a nocturnal lizard. Ecology 85: 3119–3131.

[ref96] Kearney M, Simpson SJ, Raubenheimer D, Helmuth B (2010) Modelling the ecological niche from functional traits. Philos Trans R Soc B, Biol Sci 365: 3469–3483. 10.1098/rstb.2010.0034 PMC2981966 20921046

[ref97] Kearney MR (2019) The fundamental niche concept connects individuals to populations: a comment on angilletta et al. Integr Comp Biol 59: 1509–1510. 31397868 10.1093/icb/icz147

[ref98] Kearney MR, Jusup M, McGeoch MA, Kooijman SALM, Chown SL (2021) Where do functional traits come from? The role of theory and models. Funct Ecol 35: 1385–1396.

[ref99] Kearney MR, Porter WP (2017) NichemapR—an R package for biophysical modelling: the microclimate model. Ecography 40: 664–674.

[ref100] Kessler A, Baldwin IT (2001) Defensive function of herbivore-induced plant volatile emissions in nature. Science 291: 2141. 11251117 10.1126/science.291.5511.2141

[ref101] Kiers ET, Duhamel M, Beesetty Y, Mensah JA, Franken O, Verbruggen E, Fellbaum CR, Kowalchuk GA, Hart MM, Bago A et al. (2011) Reciprocal rewards stabilize cooperation in the mycorrhizal symbiosis. Science 333: 880. 21836016 10.1126/science.1208473

[ref102] Kiers ET, Rousseau RA, West SA, Denison RF (2003) Host sanctions and the legume–rhizobium mutualism. Nature 425: 78–81. 12955144 10.1038/nature01931

[ref103] King DA, Davies SJ, Supardi MNN, Tan S (2005) Tree growth is related to light interception and wood density in two mixed dipterocarp forests of Malaysia. Funct Ecol 19: 445–453.

[ref104] King DA, Davies SJ, Tan S, Noor NSM (2006) The role of wood density and stem support costs in the growth and mortality of tropical trees. J Ecol 94: 670–680.

[ref105] Klanjšček T, Muller EB, Holden PA, Nisbet RM (2017) Host–symbiont interaction model explains non-monotonic response of soybean growth and seed production to nano-ceo2 exposure. Environ Sci Technol 51: 4944–4950. 28333444 10.1021/acs.est.6b06618

[ref106] Knops JMH, Bradley KL, Wedin DA (2002) Mechanisms of plant species impacts on ecosystem nitrogen cycling. Ecol Lett 5: 454–466.

[ref107] Kobe RK (1997) Carbohydrate allocation to storage as a basis of interspecific variation in sapling survivorship and growth. Oikos 80: 226–233.

[ref108] Kobe RK, Coats KD (1997) Models of sapling mortality as a function of growth to characterize interspecific variation in shade tolerance of eight tree species in northwestern British Colombia. Can J For Res 27: 227–236.

[ref109] Koch J, De Schamphelaere KAC (2020) Estimating inter-individual variability of dynamic energy budget model parameters for the copepod nitocra spinipes from existing life-history data. Ecol Model 431: 109091.

[ref110] Köcher P, Horna V, Leuschner C (2013) Stem water storage in five coexisting temperate broad-leaved tree species: significance, temporal dynamics and dependence on tree functional traits. Tree Physiol 33: 817–832. 23999137 10.1093/treephys/tpt055

[ref111] Kochsiek A, Tan S, Russo SE (2013) Fine root dynamics in relation to nutrients in oligotrophic bornean rain forest soils. Plant Ecol 214: 869–882.

[ref112] Kooijman SA (2001) Quantitative aspects of metabolic organization: a discussion of concepts. Philos Trans R Soc Lond B Biol Sci 356: 331–349. 11316483 10.1098/rstb.2000.0771 PMC1088431

[ref113] Kooijman SALM (1986) Population dynamics on basis of budgets. In JAJ Metz, O Diekmann, eds, The Dynamics of Physiologically Structured Populations. Springer, Berlin.

[ref114] Kooijman SALM (2010) Dynamic Budget Theory for Metabolic Organization. Cambridge University Press, Cambridge.

[ref115] Kooijman SALM (2020) The standard dynamic energy budget model has no plausible alternatives. Ecol Model 428: 109106.

[ref116] Kozlowski T, Pallardy S (1996) Growth Control in Woody Plants. Academic Press, San Diego, California.

[ref117] Kozlowski TT, Pallardy SG (2002) Acclimation and adaptive responses of woody plants to environmental stresses. Bot Rev 68: 270–334.

[ref118] Lambers H, Chapin FS III, Pons TL (1998) Plant Physiological Ecology. Springer, Berlin

[ref119] Lambers H, Poorter H (1992) Inherent variation in growth rate between higher plants: a search for physiological causes and ecological consequences. Adv Ecol Res 34: 187–261.

[ref120] Lambers H, Ribas-Carbo M (2005) Plant Respiration: From Cell to Ecosystem. Springer, The Netherlands

[ref121] Lavaud R, Filgueira R, Augustine S (2021) The role of dynamic energy budgets in conservation physiology. Conserv Physiol 9: 1-coab083. 10.1093/conphys/coab083 PMC8545044 34707875

[ref122] Lavaud R, Filgueira R, Nadeau A, Steeves L, Guyondet T (2020) A dynamic energy budget model for the macroalga *Ulva lactuca*. Ecol Model 418: 108922.

[ref123] Lavaud R, Thomas Y, Pecquerie L, Benoît HP, Guyondet T, Flye-Sainte-Marie J, Chabot D (2019) Modeling the impact of hypoxia on the energy budget of Atlantic cod in two populations of the Gulf of Saint Lawrence, Canada. J Sea Res 143: 243–253.

[ref124] Ledder G (2014) The basic dynamic energy budget model and some implications. Lett Biomath 1: 221–233.

[ref125] Ledder G, Russo SE, Muller EB, Peace A, Nisbet RM (2020) Local control of resource allocation is sufficient to model optimal dynamics in syntrophic systems. Theor Ecol 13: 481–501.

[ref126] Leigh A, Sevanto S, Close JD, Nicotra AB (2017) The influence of leaf size and shape on leaf thermal dynamics: does theory hold up under natural conditions? Plant Cell Environ 40: 237–248. 28026874 10.1111/pce.12857

[ref127] Leitner D, Klepsch S, Ptashnyk M, Marchant A, Kirk GJD, Schnepf A, Roose T (2010) A dynamic model of nutrient uptake by root hairs. New Phytol 185: 792–802. 20028467 10.1111/j.1469-8137.2009.03128.x

[ref128] Lika K, Kearney MR, Kooijman SALM (2011) The “covariation method” for estimating the parameters of the standard dynamic energy budget model ii: properties and preliminary patterns. J Sea Res 66: 278–288.

[ref129] Lima SL, Dill LM (1990) Behavioral decisions made under the risk of predation. Can J Zool 68: 619–640.

[ref130] Liu J, Zhang D, Zhou G, Duan H (2012) Changes in leaf nutrient traits and photosynthesis of four tree species: effects of elevated [co2], n fertilization and canopy positions. J Plant Ecol 5: 376–390.

[ref131] Loehle C (1988) Tree life history strategies: the role of defenses. Can J For Res 18: 209–222.

[ref132] Loehle C (1996) Optimal defensive investments in plants. Oikos 75: 299–302.

[ref133] Lorena A, Marques GM, Kooijman SALM, Sousa T (2010) Stylized facts in microalgal growth: interpretation in a dynamic energy budget context. Philos Trans R Soc B, Biol Sci 365: 3509–3521. 10.1098/rstb.2010.0101 PMC2981973 20921049

[ref134] Lucas WJ, Groover A, Lichtenberger R, Furuta K, Yadav S-R, Helariutta Y, He X-Q, Fukuda H, Kang J, Brady SM et al. (2013) The plant vascular system: evolution, development and functions. J Integr Plant Biol 55: 294–388. 23462277 10.1111/jipb.12041

[ref135] Lüttge U (2019) Plants: unitary organisms emerging from integration and self-organization of modules. In LH Wegner, U Lüttge, eds, Emergence and Modularity in Life Sciences. Springer International Publishing, Cham, pp. 171–193

[ref136] Maino JL, Kearney MR, Nisbet RM, Kooijman SALM (2014) Reconciling theories for metabolic scaling. J Anim Ecol 83: 20–29. 23668377 10.1111/1365-2656.12085

[ref137] Maire V, Wright IJ, Prentice IC, Batjes NH, Bhaskar R, van Bodegom PM, Cornwell WK, Ellsworth D, Niinemets Ü, Ordonez A et al. (2015) Global effects of soil and climate on leaf photosynthetic traits and rates. Glob Ecol Biogeogr 24: 706–717.

[ref138] Marks CO, Lechowicz MJ (2006) Alternative designs and the evolution of functional diversity. Am Nat 167: 55–66. 16475099 10.1086/498276

[ref139] Marques GM, Augustine S, Lika K, Pecquerie L, Domingos T, Kooijman SALM (2018) The AmP project: comparing species on the basis of dynamic energy budget parameters. PLoS Comput Biol 14: e1006100. 29742099 10.1371/journal.pcbi.1006100 PMC5962104

[ref140] Marschner H (1995) Mineral Nutrition in Higher Plants. Academic Press, London.

[ref141] Martin BT, Heintz R, Danner EM, Nisbet RM (2017) Integrating lipid storage into general representations of fish energetics. J Anim Ecol 86: 812–825. 28326538 10.1111/1365-2656.12667

[ref142] Matesanz S, Gianoli E, Valladares F (2010) Global change and the evolution of phenotypic plasticity in plants. Ann N Y Acad Sci 1206: 35–55. 20860682 10.1111/j.1749-6632.2010.05704.x

[ref143] McCormack ML, Dickie IA, Eissenstat DM, Fahey TJ, Fernandez CW, Guo D, Helmisaari H-S, Hobbie EA, Iversen CM, Jackson RB et al. (2015) Redefining fine roots improves understanding of below-ground contributions to terrestrial biosphere processes. New Phytol 207: 505–518. 25756288 10.1111/nph.13363

[ref144] McDowell N, Pockman WT, Allen CD, Breshears DD, Cobb N, Kolb T, Plaut J, Sperry J, West A, Williams DG et al. (2008) Mechanisms of plant survival and mortality during drought: why do some plants survive while others succumb to drought? New Phytol 178: 719–739. 18422905 10.1111/j.1469-8137.2008.02436.x

[ref145] McNickle GG, St. Clair CC, Cahill JF (2009) Focusing the metaphor: plant root foraging behaviour. Trends Ecol Evol 24: 419–426. 19409652 10.1016/j.tree.2009.03.004

[ref146] Medvigy D, Wofsy SC, Munger JW, Hollinger DY, Moorcroft PR (2009) Mechanistic scaling of ecosystem function and dynamics in space and time: ecosystem demography model version 2. J Geophys Res Biogeo 114: G01002.

[ref147] Meinzer FC, James SA, Goldstein G (2004) Dynamics of transpiration, sap flow and use of stored water in tropical forest canopy trees. Tree Physiol 24: 901–909. 15172840 10.1093/treephys/24.8.901

[ref148] Meinzer FC, James SA, Goldstein G, Woodruff D (2003) Whole-tree water transport scales with sapwood capacitance in tropical forest canopy trees. Plant Cell Environ 26: 1147–1155.

[ref149] Merow C, Smith MJ, Silander JA Jr (2013) A practical guide to maxent for modeling species’ distributions: what it does, and why inputs and settings matter. Ecography 36: 1058–1069.

[ref150] Mithöfer A, Boland W (2012) Plant defense against herbivores: chemical aspects. Annu Rev Plant Biol 63: 431–450. 22404468 10.1146/annurev-arplant-042110-103854

[ref151] Moorcroft PR, Hurtt GC, Pacala SW (2001) A method for scaling vegetation dynamics: the ecosystem demography model. Ecology 71: 557–586.

[ref152] Mounier F, Loizeau V, Pecquerie L, Drouineau H, Labadie P, Budzinski H, Lobry J (2020) Dietary bioaccumulation of persistent organic pollutants in the common sole *Solea solea* in the context of global change. Part 2: sensitivity of juvenile growth and contamination to toxicokinetic parameters uncertainty and environmental conditions variability in estuaries. Ecol Model 431: 109196.

[ref153] Muller EB (2011) Synthesizing units as modeling tool for photosynthesizing organisms with photoinhibition and nutrient limitation. Ecol Model 222: 637–644.

[ref154] Muller EB, Kooijman SA, Edmunds PJ, Doyle FJ, Nisbet RM (2009) Dynamic energy budgets in syntrophic symbiotic relationships between heterotrophic hosts and photoautotrophic symbionts. J Theor Biol 259: 44–57. 19285512 10.1016/j.jtbi.2009.03.004

[ref155] Muller EB, Lika K, Nisbet RM, Schultz IR, Casas J, Gergs A, Murphy CA, Nacci D, Watanabe KH (2019) Regulation of reproductive processes with dynamic energy budgets. Funct Ecol 33: 819–832. 32038063 10.1111/1365-2435.13298 PMC7006839

[ref156] Muller EB, Nisbet RM (2000) Survival and production in variable resource environments. Bull Math Biol 62: 1163–1189. 11127518 10.1006/bulm.2000.0203

[ref157] Nehls U, Hampp R (2000) Carbon allocation in ectomycorrhizas. Physiol Mol Plant Pathol 57: 95–100. 10.1094/MPMI.1998.11.3.167 9487692

[ref158] Nicotra AB, Atkin OK, Bonser SP, Davidson AM, Finnegan EJ, Mathesius U, Poot P, Purugganan MD, Richards CL, Valladares F et al. (2010) Plant phenotypic plasticity in a changing climate. Trends Plant Sci 15: 684–692. 20970368 10.1016/j.tplants.2010.09.008

[ref159] Nicotra AB, Davidson A (2010) Adaptive phenotypic plasticity and plant water use. Funct Plant Biol 37: 117–127.

[ref160] Niinemets Ü, Kull O (1995) Effects of light availability and tree size on the architecture of assimilative surface in the canopy of *Picea abies*: variation in needle morphology. Tree Physiol 15: 307–315. 14965954 10.1093/treephys/15.5.307

[ref161] Nobel PS (2020) Physicochemical and Environmental Plant Physiology. Academic Press, London.

[ref162] Oborny B (1991) Criticisms on optimal foraging in plants: a review. Abstracta Botanica 15: 67–76.

[ref163] Oborny B (2019) The plant body as a network of semi-autonomous agents: a review. Philos Trans R Soc London Series B, Biol Sci 374: 20180371–20180371. 10.1098/rstb.2018.0371 PMC6553591 31006361

[ref164] Obrien MJ, Leuzinger S, Philipson CD, Tay J, Hector A (2014) Drought survival of tropical tree seedlings enhanced by non-structural carbohydrate levels. Nat Clim Change 4: 710–714.

[ref165] Ogawa K (2019) Scaling relations based on the geometric and metabolic theories in woody plant species: a review. Perspect Plant Ecol Evol Syst 40: 125480.

[ref166] Paré PW, Tumlinson JH (1999) Plant volatiles as a defense against insect herbivores. Plant Physiol 121: 325. 10517823 PMC1539229

[ref167] Peay KG (2016) The mutualistic niche: mycorrhizal symbiosis and community dynamics. Annu Rev Ecol Evol Syst 47: 143–164.

[ref168] Pfab F, Brown S, Detmer AR, Baxter EC, Moeller HV, Cunning R, Nisbet RM Timescale separation and models of symbiosis: state space reduction, multiple attractors and initialization. Conserv Physiol 10: coac026. 35539007 10.1093/conphys/coac026 PMC9073712

[ref169] Pigliucci M (2003) Phenotypic integration: studying the ecology and evolution of complex phenotypes. Ecol Lett 6: 265–272.

[ref170] Plavcová L, Jansen S (2015) The role of xylem parenchyma in the storage and utilization of nonstructural carbohydrates. In U Hacke, ed, Functional and Ecological Xylem Anatomy. Springer International Publishing, Cham, pp. 209–234

[ref171] Poorter H, Evans JR (1998) Photosynthetic nitrogen-use efficiency of species that differ inherently in specific leaf area. Oecologia 116: 26–37. 28308535 10.1007/s004420050560

[ref172] Poorter H, Niklas KJ, Reich PB, Oleksyn J, Poot P, Mommer L (2012) Biomass allocation to leaves, stems and roots: meta-analyses of interspecific variation and environmental control. New Phytol 193: 30–50. 22085245 10.1111/j.1469-8137.2011.03952.x

[ref173] Pringle EG (2016) Integrating plant carbon dynamics with mutualism ecology. New Phytol 210: 71–75. 26414800 10.1111/nph.13679

[ref174] van der Putten WH, Bardgett RD, Bever JD, Bezemer TM, Casper BB, Fukami T, Kardol P, Klironomos JN, Kulmatiski A, Schweitzer JA et al. (2013) Plant–soil feedbacks: the past, the present and future challenges. J Ecol 101: 265–276.

[ref175] Récapet C, Baillif H, Buoro M (2019) Modelling Metabolism to Test Alternative Hypotheses on Intraspecific Life-History Variation, 4. Colloque d'Ecophysiologie Animale (CEPA4), Rennes, France.

[ref176] Reich PB (2014) The world-wide ‘fast–slow’ plant economics spectrum: a traits manifesto. J Ecol 102: 275–301.

[ref177] Reich PB, Ellsworth DS, Walters MB, Vose JM, Gresham C, Volin JC, Bowman WD (1999) Generality of leaf trait relationships: a test across six biomes. Ecology 80: 1955–1969.

[ref178] Reich PB, Walters MB, Tabone TJ (1989) Response of *Ulmus americana* seedlings to varying nitrogen and water status. 2 water and nitrogen use efficiency in photosynthesis. Tree Physiol 5: 173–184. 14972985 10.1093/treephys/5.2.173

[ref179] Rijkers T, Pons TL, Bongers F (2000) The effect of tree height and light availability on photosynthetic leaf traits of four Neotropical species differing in shade tolerance. Funct Ecol 14: 77–86.

[ref180] Robinson D, Hodge A, Griffiths BS, Fitter AH (1999) Plant root proliferation in nitrogen-rich patches confers competitive advantage. Proc Biol Sci 266: 431–435.

[ref181] Roy BA, Alexander HM, Davidson J, Campbell FT, Burdon JJ, Sniezko R, Brasier C (2014) Increasing forest loss worldwide from invasive pests requires new trade regulations. Front Ecol Environ 12: 457–465.

[ref182] Rüger N, Comita Liza S, Condit R, Purves D, Rosenbaum B, Visser Marco D, Wright SJ, Wirth C (2018) Beyond the fast–slow continuum: demographic dimensions structuring a tropical tree community. Ecol Lett 21: 1075–1084. 29744992 10.1111/ele.12974

[ref183] Russo SE, Jenkins KL, Wiser SK, Uriarte M, Duncan RP, Coomes DA (2010) Interspecific relationships among growth, mortality and xylem traits of woody species from New Zealand. Funct Ecol 24: 253–262.

[ref184] Russo SE, McMahon SM, Detto M, Ledder G, Wright SJ, Condit RS, Davies SJ, Ashton PS, Bunyavejchewin S, Chang-Yang C-H et al. (2021) The interspecific growth–mortality trade-off is not a general framework for tropical forest community structure. Nat Ecol Evol 5: 174–183. 33199870 10.1038/s41559-020-01340-9

[ref185] Sala A, Piper F, Hoch G (2010) Physiological mechanisms of drought-induced tree mortality are far from being resolved. New Phytol 186: 274–281. 20409184 10.1111/j.1469-8137.2009.03167.x

[ref186] Sala A, Woodruff DR, Meinzer FC (2012) Carbon dynamics in trees: feast or famine? Tree Physiol 32: 764–775. 22302370 10.1093/treephys/tpr143

[ref187] Salguero-Gómez R (2016) Applications of the fast–slow continuum and reproductive strategy framework of plant life histories. New Phytol 213: 1618–1624. 27864957 10.1111/nph.14289

[ref188] Salguero-Gómez R, Jones OR, Jongejans E, Blomberg SP, Hodgson DJ, Mbeau-Ache C, Zuidema PA, de Kroon H, Buckley YM (2016) Fast–slow continuum and reproductive strategies structure plant life-history variation worldwide. Proc Natl Acad Sci 113: 230–235. 26699477 10.1073/pnas.1506215112 PMC4711876

[ref189] Salisbury FB, Ross CW (1992) Plant Physiology. Wadsworth Publishing, Belmont

[ref190] Schimel JP, Bennett J (2004) Nitrogen mineralization: challenges of a changing paradigm. Ecology 85: 591–602.

[ref191] Schlichting CD (1986) The evolution of phenotypic plasticity in plants. Annu Rev Ecol Syst 17: 667–693.

[ref192] Schouten R, Vesk PA, Kearney MR (2020) Integrating dynamic plant growth models and microclimates for species distribution modelling. Ecol Model 435: 109262.

[ref193] Sharma S, Andrus R, Bergeron Y, Bogdziewicz M, Bragg DC, Brockway D, Cleavitt NL, Courbaud B, Das AJ, Dietze M et al. (2022) North American tree migration paced by climate in the west, lagging in the east. Proc Natl Acad Sci 119: e2116691118. 34983867 10.1073/pnas.2116691118 PMC8784119

[ref194] Silvertown J, Franco M, Harper JL (1997) Plant Life Histories: Ecology, Phylogeny and Evolution. University of Cambridge Press, Cambridge, p. 331

[ref195] Simms EL, Rausher MD (1987) Costs and benefits of plant resistance to herbivory. Am Nat 130: 570–581.

[ref196] Slot M, Krause GH, Krause B, Hernández GG, Winter K (2019) Photosynthetic heat tolerance of shade and sun leaves of three tropical tree species. Photosynth Res 141: 119–130. 30054784 10.1007/s11120-018-0563-3

[ref197] Smith AM, Stitt M (2007) Coordination of carbon supply and plant growth. Plant Cell Environ 30: 1126–1149. 17661751 10.1111/j.1365-3040.2007.01708.x

[ref198] Smith FA, Smith SE (2013) How useful is the mutualism-parasitism continuum of arbuscular mycorrhizal functioning? Plant Soil 363: 7–18.

[ref199] Smith NG, Li G, Dukes JS (2019) Short-term thermal acclimation of dark respiration is greater in non-photosynthetic than in photosynthetic tissues. AoB PLANTS 11: plz064. 10.1093/aobpla/plz064 PMC6863468 31777651

[ref200] Smith SE, Read D (2008) Mycorrhizal Symbiosis. Academic Press, London

[ref201] Smith SJ, Edmonds J, Hartin CA, Mundra A, Calvin K (2015) Near-term acceleration in the rate of temperature change. Nat Clim Change 5: 333–336.

[ref202] Soudzilovskaia NA, van der Heijden MGA, Cornelissen JHC, Makarov MI, Onipchenko VG, Maslov MN, Akhmetzhanova AA, van Bodegom PM (2015) Quantitative assessment of the differential impacts of arbuscular and ectomycorrhiza on soil carbon cycling. New Phytol 208: 280–293. 26011828 10.1111/nph.13447

[ref203] Sousa T, Domingos T, Poggiale JC, Kooijman SALM (2010) Dynamic energy budget theory restores coherence in biology. Philos Trans R Soc B, Biol Sci 365: 3413–3428. 10.1098/rstb.2010.0166 PMC2981977 20921042

[ref204] Sperry JS, Meinzer FC, McCulloh KA (2008) Safety and efficiency conflicts in hydraulic architecture: scaling from tissues to trees. Plant Cell Environ 31: 632–645. 18088335 10.1111/j.1365-3040.2007.01765.x

[ref205] Sperry JS, Smith DD, Savage VM, Enquist BJ, McCulloh KA, Reich PB, Bentley LP, von Allmen EI (2012) A species-level model for metabolic scaling in trees i. Exploring boundaries to scaling space within and across species. Funct Ecol 26: 1054–1065.

[ref206] Sperry JS, Tyree MT (1988) Mechanism of water stress-induced xylem embolism. Plant Physiol 88: 581. 16666352 10.1104/pp.88.3.581 PMC1055628

[ref207] Stearns SC (1992) The Evolution of Life Histories. Oxford University Press, Oxford

[ref208] Sterck F, Markesteijn L, Schieving F, Poorter L (2011) Functional traits determine trade-offs and niches in a tropical forest community. Proc Natl Acad Sci 108: 20627–20632. 22106283 10.1073/pnas.1106950108 PMC3251078

[ref209] Sterck F, Schieving F (2011) Modelling functional trait acclimation for trees of different height in a forest light gradient: emergent patterns driven by carbon gain maximization. Tree Physiol 31: 1024–1037. 21893522 10.1093/treephys/tpr065

[ref210] Sulis M, Couvreur V, Keune J, Cai G, Trebs I, Junk J, Shrestha P, Simmer C, Kollet SJ, Vereecken H et al. (2019) Incorporating a root water uptake model based on the hydraulic architecture approach in terrestrial systems simulations. Agric For Meteorol 269-270: 28–45.

[ref211] Sultan SE (1995) Phenotypic plasticity and plant adaptation. Acta Bot Neerl 44: 363–383.

[ref212] Tatarko AR, Knops JMH (2018) Nitrogen addition and ecosystem functioning: both species abundances and traits alter community structure and function. Ecosphere 9: e02087.

[ref213] Tedersoo L, Bahram M, Zobel M (2020) How mycorrhizal associations drive plant population and community biology. Science 367: eaba1223. 32079744 10.1126/science.aba1223

[ref214] Tedersoo L, Brundrett MC (2017) Evolution of ectomycorrhizal symbiosis in plants. In L Tedersoo, ed, Biogeography of Mycorrhizal Symbiosis. Springer International Publishing, Cham, pp. 407–467

[ref215] Thirkell TJ, Pastok D, Field KJ (2020) Carbon for nutrient exchange between arbuscular mycorrhizal fungi and wheat varies according to cultivar and changes in atmospheric carbon dioxide concentration. Glob Chang Biol 26: 1725–1738. 31645088 10.1111/gcb.14851 PMC7079082

[ref216] Tinker PB, Nye PH (2000) Solute Movement in the Rhizosphere. Oxford University Press, Oxford.

[ref217] Troost TA, Kooi BW, Kooijman SA (2005) When do mixotrophs specialize? Adaptive dynamics theory applied to a dynamic energy budget model. Math Biosci 193: 159–182. 15748728 10.1016/j.mbs.2004.06.010

[ref218] Tumlinson J, Lewis W, Vet L (1993) How parasitic wasps find their hosts. Sci Am 268: 100–106.3.8516669

[ref219] Tuomi J, Vuorisalo T (1989) Hierarchical selection in modular organisms. Trends Ecol Evol 4: 209–213. 21227352 10.1016/0169-5347(89)90075-X

[ref220] Tyree MT (1999) Water relations and hydraulic architecture. In FI Pugnaire, F Vallardes, eds, Handbook of Functional Plant Ecology. Marcel Dekker, Inc., New York, pp. 222–268

[ref221] Tyree MT (2003) Hydraulic limits on tree performance: transpiration, carbon gain and growth of trees. Trees 17: 95–100.

[ref222] Tyree MT, Ewers FW (1991) The hydraulic architecture of trees and other woody-plants. New Phytol 119: 345–360.

[ref223] Tyree MT, Sperry JS (1988) Do woody-plants operate near the point of catastrophic xylem dysfunction caused by dynamic water-stress? Answers from a model. Plant Physiol 88: 574–580. 16666351 10.1104/pp.88.3.574 PMC1055627

[ref224] Tyree MT, Sperry JS (1989) Vulnerability of xylem to cavitation and embolism. Annu Rev Plant Physiol Plant Mol Biol 40: 19–36.

[ref225] Valladares F, Matesanz S, Guilhaumon F, Araújo MB, Balaguer L, Benito-Garzón M, Cornwell W, Gianoli E, van Kleunen M, Naya DE et al. (2014) The effects of phenotypic plasticity and local adaptation on forecasts of species range shifts under climate change. Ecol Lett 17: 1351–1364. 25205436 10.1111/ele.12348

[ref226] Via S, Lande R (1985) Genotype-environment interaction and the evolution of phenotypic plasticity. Evolution 39: 505–522. 28561964 10.1111/j.1558-5646.1985.tb00391.x

[ref227] Wahid A, Gelani S, Ashraf M, Foolad MR (2007) Heat tolerance in plants: an overview. Environ Exp Bot 61: 199–223.

[ref228] Waisel Y, Eshel A, Beeckman T, Kafkafi U (2002) Plant Roots: The Hidden Half, 3rd Ed. Marcel Dekker, New York & Basel.

[ref229] Wang Y, Sperry JS, Anderegg WRL, Venturas MD, Trugman AT (2020) A theoretical and empirical assessment of stomatal optimization modeling. New Phytol 227: 311–325. 32248532 10.1111/nph.16572

[ref230] Way DA, Oren R (2010) Differential responses to changes in growth temperature between trees from different functional groups and biomes: a review and synthesis of data. Tree Physiol 30: 669–688. 20368338 10.1093/treephys/tpq015

[ref231] Way DA, Oren R, Kim H-S, Katul GG (2011) How well do stomatal conductance models perform on closing plant carbon budgets? A test using seedlings grown under current and elevated air temperatures. J Geophys Res Biogeosci 116: G04031.

[ref232] Weemstra M, Kiorapostolou N, van Ruijven J, Mommer L, de Vries J, Sterck F (2020) The role of fine-root mass, specific root length and life span in tree performance: a whole-tree exploration. Funct Ecol 34: 575–585.

[ref233] Weemstra M, Mommer L, Visser EJW, van Ruijven J, Kuyper TW, Mohren GMJ, Sterck FJ (2016) Towards a multidimensional root trait framework: a tree root review. New Phytol 211: 1159–1169. 27174359 10.1111/nph.14003

[ref251] Weemstra M, Peay KG, Davies SJ, Mohamad M, Itoh A, Tan S, Russo SE (2020) Lithological constraints on resource economies shape the mycorrhizal composition of a Bornean rain forest. New Phytol 228: 253–268. 32436227 10.1111/nph.16672

[ref234] Weiner J (2004) Allocation, plasticity and allometry in plants. Perspect Plant Ecol Evol Syst 6: 207–215.

[ref235] Weiner J, Campbell LG, Pino J, Echarte L (2009) The allometry of reproduction within plant populations. J Ecol 97: 1220–1233.

[ref236] Westoby M, Warton D, Reich PB (2000) The time value of leaf area. Am Nat 155: 649–656. 10777437 10.1086/303346

[ref237] Wiley E, Helliker B (2012) A re-evaluation of carbon storage in trees lends greater support for carbon limitation to growth. New Phytol 195: 285–289. 22568553 10.1111/j.1469-8137.2012.04180.x

[ref238] Wiley E, King CM, Landhäusser SM (2019) Identifying the relevant carbohydrate storage pools available for remobilization in aspen roots. Tree Physiol 39: 1109–1120. 31094427 10.1093/treephys/tpz051

[ref239] Williams JW, Jackson ST, Kutzbach JE (2007) Projected distributions of novel and disappearing climates by 2100 AD. Proc Natl Acad Sci 104: 5738. 17389402 10.1073/pnas.0606292104 PMC1851561

[ref240] Wink M (2010) Introduction: biochemistry, physiology and ecological functions of secondary metabolites. Annual Plant Reviews, Biochemistry of Plant Secondary Metabolism 40, 1–19.

[ref241] Wolf A, Anderegg WRL, Pacala SW (2016) Optimal stomatal behavior with competition for water and risk of hydraulic impairment. Proc Natl Acad Sci U S A 113: E7222–E7230. 27799540 10.1073/pnas.1615144113 PMC5135368

[ref242] Worthy SJ, Laughlin DC, Zambrano J, Umaña MN, Zhang C, Lin L, Cao M, Swenson NG (2020) Alternative designs and tropical tree seedling growth performance landscapes. Ecology 101: e03007. 32030743 10.1002/ecy.3007

[ref252] Wright IJ, Westoby M (2002) Leaves at low versus high rainfall: coordination of structure, lifespan and physiology. New Phytol 155: 403–416. 33873314 10.1046/j.1469-8137.2002.00479.x

[ref243] Wright IJ, Reich PB, Westoby M, Ackerly DD, Baruch Z, Bongers F, Cavender-Bares J, Chapin T, Cornelissen JHC, Diemer M et al. (2004) The worldwide leaf economics spectrum. Nature 428: 821–827. 15103368 10.1038/nature02403

[ref244] Xia M, Guo D, Pregitzer KS (2010) Ephemeral root modules in fraxinus mandshurica. New Phytol 188: 1065–1074. 21058949 10.1111/j.1469-8137.2010.03423.x

[ref245] Yamori W, Hikosaka K, Way DA (2014) Temperature response of photosynthesis in c3, c4, and cam plants: temperature acclimation and temperature adaptation. Photosynth Res 119: 101–117. 23801171 10.1007/s11120-013-9874-6

[ref246] Yoda K (1974) Three-dimensional distribution of light intensity in a tropical rain forest of West Malaysia. Jpn J Ecol 24: 247–254.

[ref247] Ziemińska K, Rosa E, Gleason SM, Holbrook NM (2020) Wood day capacitance is related to water content, wood density, and anatomy across 30 temperate tree species. Plant Cell Environ 43: 3048–3067. 32935340 10.1111/pce.13891

[ref248] Zonneveld C, Kooijman SALM (1989) Application of a dynamic energy budget model to lymnaea stagnalis (l.). Funct Ecol 3: 269–278.

[ref249] Zuleta D, Arellano G, Muller-Landau HC, McMahon SM, Aguilar S, Bunyavejchewin S, Cárdenas D, Chang-Yang C-H, Duque A, Mitre D et al. (2022) Individual tree damage dominates mortality risk factors across six tropical forests. New Phytol 233: 705–721. 34716605 10.1111/nph.17832

[ref250] Züst T, Agrawal AA (2017) Trade-offs between plant growth and defense against insect herbivory: an emerging mechanistic synthesis. Annu Rev Plant Biol 68: 513–534. 28142282 10.1146/annurev-arplant-042916-040856

