# Supplementary material for: Dynamic Energy Budget models: fertile ground for understanding resource allocation in plants in a changing world
Source: Conserv Physiol. 2022 Sep 15;10(1):coac061. doi: 10.1093/conphys/coac061 (PMC9477497; doi:10.1093/conphys/coac061)
Supplement: Russo_et_al_2022_ConsPhys_SupplApp1-2_coac061 [file russo_et_al_2022_consphys_supplapp1-2_coac061.pdf]

Supplemental Appendix 1: Analysis of the Dynamics for the Roots-Stems-Leaves Model with the Minimum Rule SU

Table 1: List of Symbols

Symbol	Meaning
Q_R, Q_S, Q_L	Production rates for roots, stems, leaves (mass/time)
R, S, L	Active biomasses of roots, stems, leaves (mass)
T_R, T_S, T_L	Turnover rates for root, stems, and leaves (mass/time)
u	Assimilation ratio $(\alpha_C S)/(\alpha_N R)$
α_C, α_N	Assimilation rate constants for C and N (1/time)
β, β_R, β_L	Leaf-Root, Stem-Root, Leaf-Stem N:C ratios (η_L/η_R, η_S/η_R, η_L/η_S)
η_R, η_S, η_L	N:C ratios for construction of roots, shoots, and leaves
ν_{RS}, ν_{SL}	N rejection from roots and stems (mass/time)
ρ_{LS}, ρ_{SR}	C rejection fluxes from leaves and stems (mass/time)

We consider an idealized plant with three components, called "roots," "stems," and "leaves", with biomasses (measured in units of carbon) $R(t)$, $S(t)$, and $L(t)$, respectively. These are abstractions of the organs responsible for assimilating water and macronutrients (roots), absorbing light and assimilating carbon into photosynthate (leaves), and transporting these materials (stems). We call the principal macronutrient "N" and the photosynthate "C" and assume water is in ample supply. The "biomass" of each component is defined to include only biologically-active tissues. Assimilated resources are used immediately to create new root, stem, and leaf biomass, with no explicit incorporation of time delays or storage. This is the same setup as in [1], except for inclusion of stems as an additional component.

The core assumptions, and the notation for state variables and for the flows of C and N, are shown in Figure 1 and listed below. (See Table 1 for a summary of notation.)

1. The components have fixed, but different, stoichiometries, with one unit of component i requiring one unit of C along with η_i units of N. Thus, C is used as a common currency for all components. The ratios of the stoichiometric factors,

$$\beta_R = \frac{\eta_S}{\eta_R} > 1, \qquad \beta_L = \frac{\eta_L}{\eta_S} > 1, \qquad \beta = \frac{\eta_L}{\eta_R} > 1, \tag{1}$$

are dimensionless measures of the relative N:C ratios in formation of a component to a lower component.

2. Resources are brought into the plant from the environment, with $\alpha_C L$ the C-assimilation rate in shoots and $\eta_R \alpha_N R$ the N-uptake rate into roots from soil.[1]

[1]The extra factor of η_R is for algebraic convenience, as it puts N assimilation into units that are directly comparable to C assimilation, given root stoichiometry.

3. Production of component biomasses occur at "synthesizing units" (SUs), which are idealized production centers that create root and shoot biomass from inputs of C and N. For this demonstration that the simple local control mechanism of Ledder et al does not work, we consider only the Liebig minimum rule.

4. Any input flux to an SU that is not used to produce new biomass constitutes a "rejection" flux that is translocated to the next component (C moves down the plant and N moves up). The C rejection fluxes from leaves and stems are ρ_{LS} and ρ_{SR}, while the N rejection fluxes from roots and stems are $\eta_S \nu_{RS}$ and $\eta_L \nu_{SL}$. Rejected C from the root SU and rejected N from leaf SU are "wasted" resources that are lost to the environment. Note that ρ_{XY} represents the potential production of Y using the C rejection flux from X; similarly, ν_{XY} represents the potential production of Y using the N rejection flux from X.

5. Component biomasses are turned over at rates T_i that are increasing functions of the relevant biomass. For this simple model, we omit possible recovery of N from lost biomass.

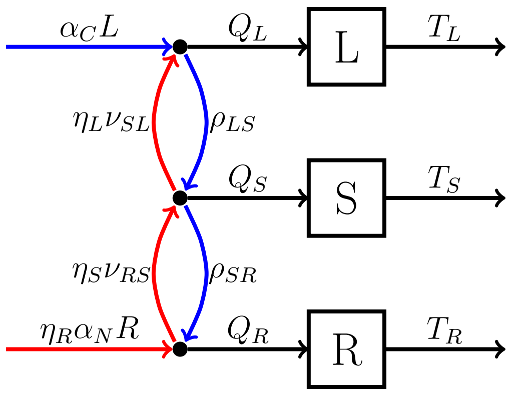

Figure 1: Resource flows in a local control model that places roots, stems, and leaves in series. The factors of η_Y in the N streams are needed because $\alpha_N R$ and ν_{XY} represent the potential production rates of the components, using C rather than N as the standard currency for organ sizes.

The model dynamics are then given by biomass balance equations

$$\frac{dR}{dt} = Q_R - T_R, \qquad \frac{dS}{dt} = Q_S - T_S, \qquad \frac{dL}{dt} = Q_L - T_L, \tag{2}$$

the SU equations

$$Q_R = \min\left(\rho_{SR}, \alpha_N R\right), \qquad Q_S = \min\left(\rho_{LS}, \nu_{RS}\right), \qquad Q_L = \min\left(\alpha_C L, \nu_{SL}\right), \tag{3}$$

and the flux balance equations

$$\rho_{SR} = \rho_{LS} - Q_S, \quad \rho_{LS} = \alpha_C L - Q_L, \quad \eta_S \nu_{RS} = \eta_R \alpha_N R - \eta_R Q_R, \quad \eta_L \nu_{SL} = \eta_S \nu_{RS} - \eta_S Q_S,$$

which we can rewrite as

$$\rho_{SR} = \rho_{LS} - Q_S, \quad \rho_{LS} = \alpha_C L - Q_L, \quad \beta_R \nu_{RS} = \alpha_N R - Q_R, \quad \beta_L \nu_{SL} = \nu_{RS} - Q_S. \tag{4}$$

The system (3–4) can be recast by using (3) to eliminate each Q_i from (4), yielding

$$\rho_{LS} = \max(\alpha_C L - \nu_{SL}, 0), \qquad \beta_R \nu_{RS} = \max(\alpha_N R - \rho_{SR}, 0), \tag{5}$$

$$\rho_{SR} = \max(\rho_{LS} - \nu_{RS}, 0), \qquad \beta_L \nu_{SL} = \max(\nu_{RS} - \rho_{LS}, 0). \tag{6}$$

The SU problem is now defined by (5)–(6) and (3).

From here, we can do a full analysis of the algebraic properties of the SU problem, as was done in Ledder et al [1], but this is not necessary to explain why the scheme does not work in this setting. Comparison of the two equations in (6) shows that ρ_{SR} and ν_{SL} cannot both be positive; that is, there cannot be both C input to the roots and N input to the leaves. Thus, there are no solutions to the SU problem that allow for simultaneous growth of roots and leaves. The full analysis shows that the only stable case, which requires $\beta_S, \beta_R > 1$, ultimately results in an equilibrium root-leaf ratio for which the C and N resource streams arrive in the correct stoichiometry for the stems, leading to maximum stem growth with no growth of either roots or leaves.

References

[1] Ledder, G., S.E. Russo, E.B. Muller, A. Peace, R. Nisbet. 2020. Local control of resource allocation is sufficient to model optimal dynamics in stable, obligate syntrophic systems.Theoretical Ecology 13:481–501

SUPPLEMENTARY APPENDIX 2: DERIVATION OF PLANT DEB MODEL

The following works out the formalism of the plant model outlined in Figure 3 and 4 in the main text. The notation closely but not entirely follows that of Kooijman (2010). In particular, the notation aims at being self-explanatory once one masters its grammar and syntax rules (see Supplementary Table 1 for rules and notation). The macrochemical equations that are implied by the model are listed in Supplementary Tables 2 and 3.

Shape considerations

Metabolism in vascular plants is organized in structures with the approximate shape of (warped) sheets (such as leaves and the cambium and phloem in dicotyledon trees) and tubes (such as stems in non-woody plants and hair roots). These two shapes share an important characteristic: their surface area to volume ratio is approximately constant if sheets do not grow in thickness and if tubes are cylinders that grow only in length. In organisms with a constant (functional) surface to volume or structural biomass ratio (so-called V1-morphs in DEB modeling), resource acquisition and allocation rates scale similarly with size. This feature greatly aids in containing model complexity; it also implies that growth is indeterminate in a constant environment. Our approach contrasts with that of Kooijman (2010), who considers plants as dynamic mixtures of shapes to account for phenomena such as self-shading. Here we refrain from including those type of self-limiting phenomena but note that those could be added to our descriptions of resource acquisition potential ('assimilation' in DEB parlance), if so desired. However, a fundamental difference between our and Kooijman's approach will remain. Whereas Kooijman's model retains a tight coupling between resource acquisition and reserve utilization rates (as both groups of rates similarly depend on plant size and shape), that coupling will be lost in ours when self-limiting features are to be included.

Shoot assimilation

With the light and dark reactions represented by a photosynthesis SU, photosynthesis is subject to two potentially limiting ‘substrates’: photosynthetically active radiation (PAR) and CO_2. With I_{PAR} and A_{chl} denoting respectively PAR irradiance and SU surface area (projected perpendicular to the irradiance) the arrival rate of PAR quanta at the photosynthetic SUs equals $A_{chl}I_{PAR}$. CO_2 comes from three sources: the atmosphere, shoot metabolism and root metabolism. Denoting the arrival fluxes of CO_2 from these sources as J_{CX}, $J_{CS\rightarrow S}$ and $J_{CR\rightarrow S}$, respectively, the net rate at

which the photosynthesis SUs are supplied with CO_2, J_C, is $J_C = J_{CX} + J_{CS\to S} + J_{CR\to S}$. $J_{CS\to S}$ and $J_{CR\to S}$ are recycling fluxes specified below.

The amounts of Rubisco and photo pigments as a fraction of leaf surface area strongly depend on the nutritional status of plants. These densities are typically low in plants growing in soils with low nitrogen content. This indicates that photosynthesis SUs cannot solely be part of structural biomass, but also comprise of shoot nitrogen rich reserve. Hence, the photosynthesis rate, J_A, is the sum of the rates at which SUs in structural biomass and those in nitrogen rich reserve produce photosynthate; these rates are J_{AV} and J_{AN}, respectively. Since photosynthesis SUs in both compartments are identical, $J_{AN} = N_N J_{AV}/N_V$, with N_N and N_V as the number of photosynthesis SUs in the nitrogen rich reserves and structural biomass, respectively. Since the numbers of SUs in each compartment are proportional to the size of its respective compartment (as implied by core DEB assumptions), $J_A = J_{AV} + J_{AN} = \left(1 + d_N m_{NS}\right) J_{AV}$. Assuming photosynthesis SUs simultaneously process substrates in parallel and with $j_* \equiv J_*/M_{VS}$,

$$j_A = \frac{1 + d_N m_{NS}}{\frac{1}{j_{Am}} + \frac{1}{j_C} + \frac{1}{y_{QA} d_{PSU} I_{PAR}} - \frac{1}{j_C + y_{QA} d_{PSU} I_{PAR}}} \quad (1)$$

in which y_{QA} is the yield of photosynthate from PAR, J_{Am} the maximum specific photosynthesis rate of SUs in structural biomass, and $d_{PSU} \equiv A_{PSU}/M_{VS}$ a constant specifying the efficiency of PAR absorption by structural shoot biomass. Rejected CO_2 is excreted at rate $j_C - j_A$ (CO_2 does not accumulate).

Photosynthate is assimilated into nitrogen rich reserves and carbon rich reserves with the SU of the former taking priority over the latter. Excess photosynthate arriving at SUs producing nitrogen reserves (ANS-SU), i.e., photosynthate that cannot be matched with sufficient nitrogen to produce nitrogen rich reserves with the proper stoichiometry, is diverted to the SU producing reduced carbon reserves. In addition to the assimilation of photosynthate, shoot nitrogen rich reserve increases due to the translocation of organic nitrogen compounds from the root. To avoid undue complexity, we assume that the elemental composition of nitrogen rich reserves in the root and shoot are similar and that the conversion efficiency of translocated organic root nitrogen into nitrogen rich shoot reserves is 100%. Then, the specific net production rate of nitrogen riche

reserves, j_{NS}, is the sum of the rate at which organic nitrogen is translocated from root to shoot ($J_{NoR\to S}$) divided by the amount of shoot structure and the specific rate of the ANS-SU, i.e.,

$$j_{NS} = \frac{J_{NoR\to S}}{M_{VS}} + j_{ANS} \tag{2}$$

With $J_{NiS\to S}$ and $J_{NiR\to S}$ as the NH_3 waste production rate due to shoot metabolism and the NH_3 translocation rate by the root, respectively, the net arrival rate of NH_3 at the ANS-SU, is $J_{NiS} = J_{NiS\to S} + J_{NiR\to S}$ (J_{NiS} and $J_{NiR\to S}$ are specified see below). The arrival rate of photosynthate at the ANS-SU, J_{AS}, includes rejected C-rich reserves from the root, $J_{ER\to S}$ (see below); accordingly, $J_{AS} = J_A + J_{ER\to S}$. Then, the specific rate of nitrogen rich reserve production in the shoot is

$$j_{ANS} = \frac{1}{\frac{1}{y_{NA} j_{AS}} + \frac{y_{NN}}{y_{NA} j_{NiS}} - \frac{y_{NN}}{y_{NA} y_{NN} j_{AS} + y_{NA} j_{NiS}}} = \frac{y_{NA}}{\frac{1}{j_A} + \frac{y_{NN}}{j_{NiS}} - \frac{y_{NN}}{y_{NN} j_A + j_{NiS}}} \tag{3}$$

in which the stoichiometric coefficients y_{NA} and y_{NN} are the yield of nitrogen rich reserves from photosynthate and the number of NH_3 molecules needed to assimilate 1 C-mole of photosynthate, respectively. The rate at which ANS-SUs reject NH_3 equals supply minus utilization: $j_{NiS} - y_{NN} j_{ANS} / y_{NA}$. A fraction $\kappa_{NiS\to R}$ returns to the root (potentially depending on hydraulic conditions), while the remainder is excreted. The (absolute) rate at which mineral N returns to the root is then $J_{NiS\to R} = \kappa_{NiS\to R} \left(j_{NiS} - y_{NN} j_{ANS} / y_{NA} \right) M_{VS}$ and the mineral N excretion rate is $\left(1 - \kappa_{NiS\to R}\right)\left(j_{NiS} - y_{NN} j_{ANS} / y_{NA} \right) M_{VS}$ (e.g. evaporation through leaves).

The specific rate at which photosynthate is rejected by ANS-SUs equals $j_{AS} - j_{ANS} / y_{NA}$. Thus, the specific rate at which carbohydrates are produced, j_{ES}, is

$$j_{ES} = y_E \left(j_A - \frac{j_{ANS}}{y_{NA}} \right) \tag{4}$$

with y_E representing the conversion efficiency of photosynthate to carbon reserve.

Shoot reserve dynamics and catabolism

The dynamics of the nitrogen and carbon reserve densities follow from core DEB assumptions and are given by

$$\frac{dm_{NS}}{dt} = j_{NS} + \kappa_{NS}\left(\left(k_{NS} - j_{VS}\right)m_{NS} - \left(1 - y_{US}\right)j_{US}\right) - k_{NS}m_{NS} \tag{5}$$

$$\frac{dm_{ES}}{dt} = j_{ES} + \kappa_{ES}\left(\left(k_{ES} - j_{VS}\right)m_{ES} - y_{US}j_{US}\right) - k_{ES}m_{ES} \tag{6}$$

in which k_{NS} and k_{ES} are the turn-over rates of nitrogen-rich and carbon reserve, respectively, and j_{US} is the specific rate at which merged reserves are utilized by the shoot catabolic SU (US-SU) (see next). The middle terms in these equations reflect that reserve mobilization may exceed the stoichiometric requirements for catabolism. A fraction of those excesses, κ_{NS} and κ_{ES}, return to the corresponding reserve pools, while the remainder is excreted and/or translocated to the root (see below); those fractions may be functions of hydraulic quantities that are not considered here. The term $\left(k_{*S} - j_{VS}\right)m_{*S}$ represents the specific mobilization rate of reserve '*' and follows from

$$\frac{dM_{*S}}{dt} = M_{VS}\frac{dm_{*S}}{dt} - m_{*S}\frac{dM_{VS}}{dt} = M_{VS}\frac{dm_{*S}}{dt} - m_{*S}J_{VS} \tag{7}$$

Accordingly, j_{US} is

$$j_{US} = \frac{1}{\dfrac{y_{US}}{\left(k_{ES} - j_{VS}\right)m_{ES}} + \dfrac{\left(1 - y_{US}\right)}{\left(k_{NS} - j_{VS}\right)m_{NS}} - \dfrac{y_{US}\left(1 - y_{US}\right)}{\left(1 - y_{US}\right)\left(k_{ES} - j_{VS}\right)m_{ES} + y_{US}\left(k_{NS} - j_{VS}\right)m_{NS}}} \tag{8}$$

with y_{US} as the amount of carbohydrates needed by US-SUs to form a generalized compound with fixed stoichiometric composition for catabolism.

Excess nitrogen rich and carbon reserve rejected by the shoot catabolic SU may be in part returned to their respective reserve pool, excreted or translocated to the root. The absolute rates at which the shoot translocates nitrogen rich and carbon reserve to the root are

$$J_{NS\to R} = M_{VS}\kappa_{NS\to R}\left(1 - \kappa_{NS}\right)\left(\left(k_{NS} - j_{VS}\right)m_{NS} - \left(1 - y_{US}\right)j_{US}\right) \tag{9}$$

$$J_{ES\to R} = M_{VS}\kappa_{ES\to R}\left(1 - \kappa_{ES}\right)\left(\left(k_{ES} - j_{VS}\right)m_{ES} - y_{US}j_{US}\right) \tag{10}$$

where $\kappa_{ES\to R}$ and $\kappa_{NS\to R}$ are the fractions of the rejection fluxes that are translocated with the remainder being excreted. Those fractions may be functions of hydraulic conditions and/or under control of the microbiome.

The catabolic flux splits in two. Under non-starvation conditions, a fraction κ of the utilization flux is committed to somatic functions, while the remainder is used for development and sexual reproduction (adults only). With animals, κ is usually considered constant, but like the partitioning factors quantifying recycling of rejected reserves, it may be a function of environmental factors, in addition to the state of maturity and/or reproductive buffer. Maintenance demands are debited first from each split utilization flux; the remainder is used for growth (somatic fraction of utilization flux) and development and reproduction (non-somatic fraction of utilization flux). Somatic growth is the increase in structural biomass, which is

$$\frac{dM_{VS}}{dt} = y_{VS}\left(\kappa j_{US} - j_{MS}\right) M_{VS} = j_{VS} M_{VS} \tag{11}$$

in which y_{VS} and j_{MS} are the growth efficiency and the specific maintenance rate, respectively. The part of the specific catabolic flux that is committed to maturation or reproduction is $\left(1-\kappa\right) j_{US} - j_{JS}$. Given the plasticity observed in plants, it is not obvious what bioenergetically related quantities may determine the transition of a juvenile into a sexually reproducing adult. In the standard DEB model for animals, juveniles become adults once a certain amount of energy has been spent on maturation. It is not clear how this would translate into the current context, as plants require an external stimulus, such as a cold period, to initiate the development of reproductive organs. Lacking a stimulus, a maturing individual in the standard DEB model is faced with the problem what to do with the resources once devoted to maturation. Inspired by the observation that apical meristems progressively lose global control over differentiation processes as the plant grows, we take a threshold amount of structural biomass at the time of the transition, M_D, as the cue for becoming sexually mature, in addition to meeting external stimuli (lacking stimuli, the plant continue to invest in maturation). Then, the rate at which resources are committed to reproduction, J_F, are

$$J_F = \begin{cases} 0 & \text{if } M_D > M_{VS} \\ y_F\left(1-\kappa\right) j_{US} M_{VS} - j_{MS} M_D & \text{if } M_D < M_{VS} \end{cases} \tag{12}$$

Shoot production of xylem and minerals

DEB fluxes can produce two types of products: biomaterials and minerals. Biomaterials include cellulose, lignin and some secondary metabolites, among other products that cannot be re-used for metabolism. We are particularly interested in quantifying the amount of xylem (in sapwood), as this quantity determines the hydraulic traits of a plant and, therefore, its capacity to assimilate CO_2, and soil nutrients, as well as translocate resources between shoot and root. Thus, this quantity plays a vital role in the future linking of DEB and hydraulic models. The minerals of direct interest are recyclable waste products, notably CO_2 and NH_3. The key to specifying product fluxes is that any DEB flux may only release products at a rate proportional to its own magnitude, i.e., compounds involved in a DEB flux are consumed or produced in fixed stoichiometric quantities. Hence, the formulations of product formation rates do not require additional assumptions but are calculated via a bookkeeping exercise.

For simplicity, we assume that growth and somatic maintenance are the only DEB fluxes contribute to cellulose, lignin and other compounds contributing to the physical integrity of a plant. Linking cellulose and lignin production to growth is obviously necessary, but it is not sufficient, as it would imply a constant cellulose and lignin to structural biomass ratio (in absence of herbivory and leaf senescence). Adding somatic maintenance as a (possibly minor) contributor of cellulose and lignin production yields flexibility and would, for instance, predict higher cellulose and lignin to structural biomass ratios in trees growing under harsher conditions. Furthermore, we assume that biomaterials produced during growth and somatic maintenance are similar in composition. Accordingly, the biomaterials production rate in the shoot, J_{PS}, is

$$J_{PS} = y_{PVS} J_{VS} + y_{PMS} J_{MS} = \left(y_{PVS} j_{VS} + y_{PMS} j_{MS} \right) M_{VS} \tag{13}$$

in which y_{PGS} and y_{PMS} are the yields of biomaterials in growth and maintenance processes, respectively. Assuming that a constant fraction κ_{XS} of biomaterials produced in the shoot are used in the formation of xylem, the dynamics of xylem in sapwood are

$$J_{XS} = \kappa_{XS} J_{PS} - \Sigma_S \tag{14}$$

in which Σ_S is function representing the rate of transition from sapwood to heartwood, among other loss factors; we leave this function unspecified for now.

The calculation of recyclable CO_2 and NH_3 production is a tedious bookkeeping exercise. Their specific production rates in juveniles are

$$j_{CS\to S} = \frac{1-y_{NA}}{y_{NA}} j_{ANS} + \frac{1-y_E}{y_E} j_{ES} + \left(1-y_{PMS}\right) j_{MS} + \frac{1-y_{VS}-y_{PVS}}{y_{VS}} j_{VS} + \left(1-\kappa\right) j_{US} \tag{17}$$

$$j_{NiS\to S} = y_{NMS} j_{MS} + y_{NDS}\left(1-\kappa\right) j_{US} \tag{18}$$

and in adults

$$\begin{aligned} j_{CS\to S} &= \frac{1-y_{NA}}{y_{NA}} j_{ANS} + \frac{1-y_E}{y_E} j_{ES} + \left(1-y_{PMS}\right) j_{MS} + \frac{1-y_{VS}-y_{PVS}}{y_{VS}} j_{VS} + \\ &\quad \frac{j_{MS}M_D}{M_{VS}} + \left(1-y_F\right)\left(\left(1-\kappa\right) j_{US} - \frac{j_{MS}M_D}{M_{VS}}\right) \\ &= \frac{1-y_{NA}}{y_{NA}} j_{ANS} + \frac{1-y_E}{y_E} j_{ES} + \left(1-y_{PMS}\right) j_{MS} + \frac{1-y_{VS}-y_{PMS}}{y_{VS}} j_{VS} + \\ &\quad \frac{y_F j_{MS} M_D}{M_{VS}} + \left(1-y_F\right)\left(1-\kappa\right) j_{US} \end{aligned} \tag{19}$$

$$\begin{aligned} j_{NiS\to S} &= j_{MS}\left(y_{NMS} + \frac{M_D}{M_{VS}} y_{NDS}\right) + y_{NF}\left(\left(1-\kappa\right) j_{US} - \frac{j_{MS}M_D}{M_{VS}}\right) \\ &= j_{MS}\left(y_{NMS} + \left(y_{NDS}-y_{NF}\right)\frac{M_D}{M_{VS}}\right) + y_{NF}\left(1-\kappa\right) j_{US} \end{aligned} \tag{20}$$

respectively. The yield coefficients y_{**} can be calculated from the macrochemical reaction equations in Table A2 and are listed in Table A4.

ROOT METABOLISM

Nitrogen uptake from soil

Soil nitrogen resources are NH_3 and NO_3^-. NO_3^- can accumulate to significant levels in vacuoles, suggesting that NO_3^- reserve dynamics should be part of the model. This would complicate matters considerably with likely little gain for most purposes, especially given that nitrogen translocation between root and shoot is generally in the form of amides (perhaps excluding translocation at high soil NO_3^- load conditions). Therefore, we assume a parallel processing SU for the uptake and reduction of NO_3^- into NH_3 with C reserves translocated from the shoot as complementary substrate supplying the energy needed to drive those processes. Costs for NH_3 are assumed negligible.

The specific rate at which roots take up NH_3 from soil, j_{Nred}, is

$$j_{Nred} = j_{Nredm} \frac{J_{NredT}}{J_{NredT} + j_{Nredm} M_{VR}} \tag{21}$$

with J_{NredT} as the rate at which soil NH_3 arrives at uptake SU and j_{Nredm} as the maximum specific NH_3 uptake rate.

The specific rate at which roots take up and transform soil NO_3^-, J_{Nox}, is

$$j_{Nox} = \frac{1}{\frac{1}{j_{Noxm}} + \frac{M_{VR}}{J_{NoxT}} + \frac{M_{VR}}{y_{NoxA} J_{ES\to R}} - \frac{M_{VR}}{J_{NoxT} + y_{NoxA} J_{ES\to R}}} \tag{22}$$

with J_{NoxT} as the arrival rate of soil NO_3^-, j_{Noxm} the maximum specific NO_3^- uptake and reduction rate, and y_{NoxA} representing the C reserve requirements for nitrate reduction.

The specific net rate at which NH_3 is available for assimilation in the root is the sum of soil uptake, recycling and translocation from shoot,

$$j_{NiR} = j_{Nred} + j_{Nox} + j_{NiR\to R} + \frac{J_{NiS\to R}}{M_{VR}} \tag{23}$$

The specific rate at which translocated C reserves after nitrate reduction are available for assimilation is

$$j_{AR} = \frac{J_{ES\to R}}{M_{VR}} - \frac{j_{Nox}}{y_{NoxA}} \tag{24}$$

The remainder of the root model is analogous to that of the shoot (minus reproduction), with the terminal 'S' in symbols identifying shoot quantities being replaced by 'R'. Therefore, we list the equations below without further elaboration.

Assimilation

$$j_{ANR} = \frac{1}{\frac{1}{y_{NA} j_{AR}} + \frac{y_{NN}}{y_{NA} j_{NiR}} - \frac{y_{NN}}{y_{NA} y_{NN} j_{AR} + y_{NA} j_{NiR}}} = \frac{y_{NA}}{\frac{1}{j_{AR}} + \frac{y_{NN}}{j_{NiR}} - \frac{y_{NN}}{y_{NN} j_{AR} + j_{NiR}}} \tag{25}$$

$$J_{NiR\to S} = \left(j_{NiR} - y_{NN} j_{ANR} / y_{NA} \right) M_{VR} \; J_{NiR\to S} = \kappa_{NiR\to S} \left(j_{NiR} - y_{NN} j_{ANR} / y_{NA} \right) M_{VR} \tag{26}$$

$$j_{ER} = y_E \left(j_{AR} - \frac{j_{ANR}}{y_{NA}} \right) \quad (27)$$

Root reserve dynamics

$$\frac{dm_{NR}}{dt} = j_{ANR} + \kappa_{NR}\left(\left(k_{NR} - j_{VR}\right)m_{NR} - \left(1 - y_{UR}\right)j_{UR}\right) - k_{NR}m_{NR} \quad (28)$$

$$\frac{dm_{ER}}{dt} = j_{ER} + \kappa_{ER}\left(\left(k_{ER} - j_{VR}\right)m_{ER} - y_{UR}j_{UR}\right) - k_{ER}m_{ER} \quad (29)$$

Root catabolism

$$j_{UR} = \frac{1}{\frac{y_{UR}}{\left(k_{ER} - j_{VR}\right)m_{ER}} + \frac{\left(1 - y_{UR}\right)}{\left(k_{NR} - j_{VR}\right)m_{NR}} - \frac{y_{UR}\left(1 - y_{UR}\right)}{\left(1 - y_{UR}\right)\left(k_{ER} - j_{VR}\right)m_{ER} + y_{UR}\left(k_{NR} - j_{VR}\right)m_{NR}}} \quad (30)$$

Fractions κ_{NR} and κ_{ER} of respectively, rejected N and C reserves return to corresponding reserve boxes, while the remainders are translocated to the shoot and/or excreted into the rhizosphere. With $\kappa_{NoR \to S}$ and $\kappa_{ER \to S}$ as the fractions of those remainders translocated to the root, the absolute translocation rates of N and C reserves are, respectively,

$$J_{NR \to S} = M_{VR}\kappa_{NoR \to S}\left(1 - \kappa_{NR}\right)\left(\left(k_{NR} - j_{VR}\right)m_{NR} - \left(1 - y_{UR}\right)j_{UR}\right) \quad (31)$$

$$J_{ER \to S} = M_{VR}\kappa_{ER \to S}\left(1 - \kappa_{ER}\right)\left(\left(k_{ER} - j_{VR}\right)m_{ER} - y_{UR}j_{UR}\right) \quad (32)$$

and the corresponding excretion fluxes are

$$J_{NR \to excr} = M_{VR}\left(1 - \kappa_{NoR \to S}\right)\left(1 - \kappa_{NR}\right)\left(\left(k_{NR} - j_{VR}\right)m_{NR} - \left(1 - y_{UR}\right)j_{UR}\right) \quad (33)$$

$$J_{ER \to excr} = M_{VR}\left(1 - \kappa_{ER \to S}\right)\left(1 - \kappa_{ER}\right)\left(\left(k_{ER} - j_{VR}\right)m_{ER} - y_{UR}j_{UR}\right) \quad (34)$$

Root growth

$$\frac{dM_{VR}}{dt} = y_{VR}\left(\kappa j_{UR} - j_{MR}\right)M_{VR} = j_{VR}M_{VR} \quad (35)$$

Root production of xylem and minerals

$$J_{PR} = y_{PVR}J_{VR} + y_{PMR}J_{MR} = \left(y_{PVR}j_{VR} - y_{PMR}j_{MR}\right)M_{VR} \quad (36)$$

$$J_{XR} = \kappa_{XR}J_{PR} - \Sigma_{R} \quad (37)$$

$$J_{CR\to S} = \kappa_{CR\to S}M_{VR}\left(\frac{1-y_{NA}}{y_{NA}}j_{NA} + \frac{1-y_{E}}{y_{E}}j_{ER} + \left(1-y_{PMR}\right)j_{MR} + \frac{1-y_{VR}-y_{PMR}}{y_{VR}}j_{VR} + j_{MR} + \left(1-\kappa\right)j_{UR}\right) \quad (38)$$

$$j_{NiR\to R} = y_{NMR}j_{MR} + y_{NDR}\left(1-\kappa\right)j_{UR} \quad (39)$$

Literature Cited

Kooijman SALM (2010) Dynamic budget theory for metabolic organization. Cambridge University Press.

Supplementary Table 1: Notational conventions and symbols

Grammar and syntax rules:

- Recurring letters in subscripts relating to
  - Biomass pools
    - V: structural biomass
    - E: C reserves (*cf.* energy)
    - N: nitrogen rich reserves (also nitrogen in general)
    - F: reproductive matter (*cf.* fertility)
    - P: products, non-metabolizable biomaterials
  - Unit/ compartment
    - X: environment (atmosphere and/or soil)
    - S: shoot
    - R: root;
    - T: soil (from ‘terre’)
  - Process
    - A: assimilation (photosynthesis, N rich reserve synthesis)
    - D: maturation
    - J: maturity maintenance
    - M: somatic maintenance
    - U: catabolism (utilization of reserves)

- Compounds (not forming pools) and elements
    - C: CO_2
    - H: hydrogen atom or H_2O
    - N: nitrogen atom or NH_3 (in stoichiometric quantities)
    - Ni: inorganic nitrogen (in fluxes)
    - No: organic nitrogen
    - O: oxygen atom or O_2

- J_*: absolute flux of compound * (mol/time)
- j_*: specific flux of compound * (mol/ mol C of structural biomass/ time)
- $J_{AB \to C}$: translocation flux of compound A from unit B to unit C
- M_{AB}: amount of generalized compound A; lower case *m* refers to a density of generalized compound in structural biomass
- y_{ABC}: stoichiometric conversion factor for compound A. Interpretation depends on context. Generally, read as "yield/ requirement of compound A from/for compound B (or during process B) in unit C ". C is omitted when unit is obvious or when the yield has the same value in shoot and root. B is omitted when the yield can be interpreted as a growth or assimilation efficiency.

Symbol	Interpretation	Units
A_{PSU}	photon capturing surface area of a photosynthesis SU	m^{-2}
d_N	ratio of densities of photosynthesizing SUs in reserves and structure	–
d_{PSU}	PAR adsorption efficiency	–
I_{PAR}	Irradiance (photosynthetically active radiation)	mol quanta. m^{-2}. day^{-1}
j_A	specific photosynthate production rate	mol C. mol C^{-1}. day^{-1}
j_{ANS}	specific production rate of nitrogen rich reserves from photosynthate	mol C. mol C^{-1}. day^{-1}
j_{ASm}	maximum specific photosynthesis rate of shoot structural biomass	mol C. mol C^{-1}. day^{-1}

j_{ES}	specific rate of C reserves formation in shoot	mol C. mol C^{-1}. day^{-1}
j_{C}	specific arrival rate of CO_2 at photosynthesis SU	mol C. mol C^{-1}. day^{-1}
j_{JS}	specific maturity maintenance rate of shoot	mol C. mol C^{-1}. day^{-1}
j_{MS}	specific somatic maintenance rate of shoot	mol C. mol C^{-1}. day^{-1}
j_{Nred}	specific NH_3 uptake rate by root	mol N. mol C^{-1}. day^{-1}
j_{Nredm}	maximum specific NH_3 uptake rate by root	mol N. mol C^{-1}. day^{-1}
j_{NiS}	specific arrival rate of inorganic N at SUs producing N rich reserves	mol N. mol C^{-1}. day^{-1}
j_{Noxm}	maximum specific NO_3^- uptake and reduction rate	mol N. mol C^{-1}. day^{-1}
j_{NS}	specific net formation rate of nitrogen rich reserves	mol C. mol C^{-1}. day^{-1}
j_{US}	specific utilization rate of reserves for shoot catabolism	mol C. mol C^{-1}. day^{-1}
j_{VS}	specific growth rate of shoot	mol C. mol C^{-1}. day^{-1}
J_{A}	photosynthate production rate	mol C. day^{-1}
J_{AN}	photosynthate production rate by SUs that are part of structural biomass	mol C. day^{-1}
J_{AV}	photosynthate production rate by SUs that are part of nitrogen rich reserves	mol C. day^{-1}
J_{C}	arrival rate of CO_2 at photosynthesis SU	mol C. day^{-1}
J_{CX}	arrival rate of atmospheric CO_2 at photosynthesis SU	*mol C. day^{-1}*
$J_{CS\to S}$	arrival rate of CO_2 from shoot metabolism at photosynthesis SU	mol C. day^{-1}
$J_{CR\to S}$	arrival rate of CO_2 from root metabolism at photosynthesis SU	mol C. day^{-1}
$J_{ES\to R}$	translocation rate of C reserves from shoot to root	mol C. day^{-1}
J_{FS}	biomaterial production rate in shoot	mol C. day^{-1}

J_{JS}	maturity maintenance rate of shoot	mol C. day^{-1}
J_{MS}	somatic maintenance rate of shoot	mol C. day^{-1}
$J_{NiR \to S}$	translocation rate of inorganic nitrogen from root to shoot	mol N. day^{-1}
J_{NiS}	arrival rate of inorganic nitrogen at SU producing N rich reserves in shoot	mol N. day^{-1}
$J_{NiS \to S}$	production rate of recyclable inorganic nitrogen in shoot	mol N. day^{-1}
J_{NoxT}	arrival rate of NO_3^- at uptake SU at root-soil interface	mol C. day^{-1}
J_{NredT}	arrival rate of NH_3 at uptake SU at root-soil interface	mol C. day^{-1}
$J_{NoR \to S}$	translocation rate of organic nitrogen from root to shoot	mol C. day^{-1}
J_F	rate at which catabolites are allocated to reproduction	mol C. day^{-1}
k_{ES}	turn-over rate of C reserves in shoot	day^{-1}
k_{ER}	turn-over rate of C reserves in root	day^{-1}
k_{NR}	turn-over rate of N rich reserves in root	day^{-1}
m_{ES}	density of C rich reserves in shoot	mol C. mol C^{-1}
m_{NS}	density of N rich reserves in shoot	mol C. mol C^{-1}
m_{ER}	density of C rich reserves in root	mol C. mol C^{-1}
m_{NR}	density of N rich reserves in root	mol C. mol C^{-1}
M_D	shoot structural biomass at reaching maturation	mol C
M_{VS}	structural biomass of shoot	mol C
M_{VR}	structural biomass of root	mol C
$n_{H,FS}$	molar H:C ratio in biomaterials produced in shoot	mol H. mol C^{-1}

$n_{O,FS}$	molar O:C ratio in biomaterials produced in shoot	mol O. mol C^{-1}
$n_{N,FS}$	molar N:C ratio in biomaterials produced in shoot	mol N. mol C^{-1}
$n_{H,N}$	molar H:C ratio in N rich reserves	mol H. mol C^{-1}
$n_{O,N}$	molar O:C ratio in N rich reserves	mol O. mol C^{-1}
$n_{N,N}$	molar N:C ratio in N rich reserves	mol N. mol C^{-1}
$n_{H,RS}$	molar H:C ratio in reproductive matter	mol H. mol C^{-1}
$n_{O,RS}$	molar O:C ratio in reproductive matter	mol O. mol C^{-1}
$n_{N,RS}$	molar N:C ratio in reproductive matter	mol N. mol C^{-1}
$n_{H,US}$	molar H:C ratio in shoot catabolites	mol H. mol C^{-1}
$n_{O,US}$	molar O:C ratio in shoot catabolites	mol O. mol C^{-1}
$n_{N,US}$	molar N:C ratio in shoot catabolites	mol N. mol C^{-1}
$n_{H,VS}$	molar H:C ratio in shoot structural biomass	mol H. mol C^{-1}
$n_{O,VS}$	molar O:C ratio in shoot structural biomass	mol O. mol C^{-1}
$n_{N,VS}$	molar N:C ratio in shoot structural biomass	mol N. mol C^{-1}
y_E	yield of C reserves from photosynthate (assimilation efficiency C reserves)	mol C. mol C
y_{PVS}	yield of biomaterials due to growth	mol C. mol C
y_{PVS}	yield of biomaterials due to somatic maintenance	mol C. mol C
y_{HDS}	yield of water during maturity maintenance and maturation in shoot	mol H_2O. mol C^{-1}
y_{HMS}	yield of water during somatic maintenance in shoot	mol H_2O. mol C^{-1}
y_{HVS}	yield of water during growth	mol H_2O. mol C^{-1}

y_{HRS}	yield of water during production of reproductive matter	mol H_2O. mol C^{-1}
y_{HN}	yield of water during assimilation of photosynthate (into N rich reserves)	mol H_2O. mol C^{-1}
y_{HUS}	requirement of water in shoot catabolism	mol H_2O. mol C^{-1}
y_{NMS}	yield of recyclable N during maturity maintenance and maturation	mol N. mol C^{-1}
y_{NA}	yield of N rich reserves from photosynthate (assimilation efficiency N reserves)	mol C. mol C^{-1}
y_{NMS}	yield of recyclable N during somatic maintenance	mol N. mol C^{-1}
y_{NN}	requirement of inorganic N for assimilation of photosynthate (into N rich reserves)	mol N. mol C^{-1}
y_{NoxA}	requirement of C reserve for nitrate reduction	mol C. mol N^{-1}
y_{NRS}	yield of recyclable N during formation of reproductive matter	mol N. mol C^{-1}
y_{NVS}	yield of recyclable N during growth	mol N. mol C^{-1}
y_{ODS}	requirement of oxygen for shoot maturity maintenance and maturation	mol O_2. mol C^{-1}
y_{OMS}	requirement of oxygen for shoot somatic maintenance	mol O_2. mol C^{-1}
y_{ON}	requirement of oxygen for assimilation of photosynthate (into N rich reserves)	mol O_2. mol C^{-1}
y_{ORS}	requirement of oxygen for production of reproductive matter	mol O_2. mol C^{-1}
y_{QA}	yield of photosynthate from captured PAR	mol C. mol $quanta^{-1}$
y_{US}	requirement of C reserves in shoot catabolism	mol C. mol C^{-1}
y_{VS}	yield of shoot structural biomass from catabolites	mol C. mol C^{-1}
κ	fraction catabolic flux to shoot growth and maintenance	-
κ_{ES}	fraction of C reserves returning to C reserves upon rejection by shoot catabolic SU	-
κ_{NS}	fraction of N rich reserves returning to N rich reserves upon rejection by shoot catabolic SU	-

Supplementary Table 2: Macrochemical reaction equations shoot.

Photosynthesis:

$$CO_2 + H_2O \longrightarrow CH_2O + O_2$$

Formation of N-rich reserves:

$$CH_2O + y_{ON}O_2 + y_{NN}NH_3 \longrightarrow y_{NA}CH_{n_{H,N}}O_{n_{O,N}}N_{n_{N,N}} + (1 - y_{NA})CO_2 + y_{HN}H_2O$$

Formation of carbohydrate reserves:

$$CH_2O + (1 - y_E)O_2 \longrightarrow y_E CH_{5/3}O_{5/6} + (1 - y_E)CO_2 + \left(1 - \tfrac{5}{6}y_E\right)H_2O$$

Mobilization of reserves for catabolism ($n_{*,US}$ are not free parameters$^{)}$:

$$y_{US}CH_{5/3}O_{5/6} + (1 - y_{US})CH_{n_{H,N}}O_{n_{O,N}}N_{n_{N,N}} + y_{HUS}H_2O \longrightarrow CH_{n_{H,US}}O_{n_{O,US}}N_{n_{N,US}}$$

Somatic maintenance (including potential production of skeletal C (F) - (hemi)cellulose, lignin):

$$CH_{n_{H,US}}O_{n_{O,US}}N_{n_{N,US}} + y_{OMS}O_2 \longrightarrow y_{PMS}CH_{n_{H,PS}}O_{n_{O,PS}} + (1 - y_{PMS})CO_2 + y_{NMS}NH_3 + y_{HMS}H_2O$$

Growth (including potential production of skeletal C (F) - (hemi)cellulose, lignin):

$$CH_{n_{H,US}}O_{n_{O,US}}N_{n_{N,US}} + y_{OVS}O_2 \longrightarrow y_{VS}CH_{n_{H,VS}}O_{n_{O,VS}}N_{n_{N,VS}} + y_{PVS}CH_{n_{H,PS}}O_{n_{O,PS}} + (1 - y_{VS} - y_{PVS})CO_2 + y_{NVS}NH_3 + y_{HVS}H_2O$$

Development and maturity maintenance:

$$CH_{n_{H,US}}O_{n_{O,US}}N_{n_{N,US}} + y_{ODS}O_2 \longrightarrow CO_2 + y_{NDS}NH_3 + y_{HDS}H_2O$$

Reproduction (fibrous material included in generalized composition of reproductive matter):

$$CH_{n_{H,US}}O_{n_{O,US}}N_{n_{N,US}} + y_{OF}O_2 \longrightarrow y_F CH_{n_{H,F}}O_{n_{O,F}}N_{n_{N,F}} + (1 - y_F)CO_2 + y_{NF}NH_3 + y_{HF}H_2O$$

Table A3: Macrochemical reaction equations root.

Conversion nitrate to ammonia:

$$CH_2O+\left(1-2y_{NoxA}\right)O_2+y_{NoxA}\left(H^+ +NO_3^-\right)\longrightarrow CO_2+\left(1-y_{NoxA}\right)H_2O+y_{NoxA}NH_3$$

Formation of N-rich reserves: same as in shoot

Formation of carbohydrate reserves: same as in shoot

Mobilization of reserves for catabolism:

$$y_{UR}CH_{5/3}O_{5/6}+\left(1-y_{UR}\right)CH_{n_{H,N}}O_{n_{O,N}}N_{n_{N,N}}+y_{HUR}H_2O\longrightarrow CH_{n_{H,UR}}O_{n_{O,UR}}N_{n_{N,UR}}$$

Somatic maintenance (including potential production of skeletal C (F) - (hemi)cellulose, lignin):

$$CH_{n_{H,UR}}O_{n_{O,UR}}N_{n_{N,UR}}+y_{OMR}O_2\longrightarrow y_{PMR}CH_{n_{H,PR}}O_{n_{O,PR}}+\left(1-y_{PMR}\right)CO_2+y_{NMR}NH_3+y_{HMR}H_2O$$

Growth (including potential production of skeletal C (F) - (hemi)cellulose, lignin):

$$CH_{n_{H,UR}}O_{n_{O,UR}}N_{n_{N,UR}}+y_{OVR}O_2\longrightarrow y_{VR}CH_{n_{H,VR}}O_{n_{O,VR}}N_{n_{N,VR}}+y_{PVR}CH_{n_{H,PR}}O_{n_{O,PR}}+\left(1-y_{VR}-y_{PVR}\right)CO_2+y_{NVR}NH_3+y_{HVR}H_2O$$

Development and maturity maintenance:

$$CH_{n_{H,UR}}O_{n_{O,UR}}N_{n_{N,UR}}+y_{ODR}O_2\longrightarrow CO_2+y_{NDR}NH_3+y_{HDR}H_2O$$

Supplementary Table 3: Mineral yield coefficients

Mineral yield coefficients of photosynthesis, N-rich reserves and carbohydrate formation in shoots

$$\begin{bmatrix} y_{CA} \\ y_{HA} \\ y_{OA} \\ y_{NA} \end{bmatrix} = \begin{bmatrix} 1 \\ 1 \\ 1 \\ 0 \end{bmatrix} \quad \begin{bmatrix} y_{CN} \\ y_{HN} \\ y_{ON} \\ y_{NN} \end{bmatrix} = \begin{bmatrix} 1 - y_{NA} \\ 1 + 1.5 n_{N,N} - 0.5 y_{NA} n_{H,N} \\ 1 + 0.75 n_{N,N} + \left(0.5 n_{O,N} - 1 - 0.25 n_{H,N}\right) y_{NA} \\ n_{N,N} \end{bmatrix} \quad \begin{bmatrix} y_{CE} \\ y_{HE} \\ y_{OE} \\ y_{NE} \end{bmatrix} = \begin{bmatrix} 1 - y_E \\ 1 - 0.833 y_E \\ 1 - y_E \\ 0 \end{bmatrix}$$

Mineral yield coefficients shoot catabolism

$$\begin{bmatrix} y_{CUS} \\ y_{HUS} \\ y_{OUS} \\ y_{NUS} \end{bmatrix} = \begin{bmatrix} 0 \\ 0.5 n_{H,US} - 0.5 n_{H,N} - \left(0.833 - 0.5 n_{H,N}\right) y_{US} \\ 0 \\ 0 \end{bmatrix}$$

Mineral yield coefficients shoot maintenance

$$\begin{bmatrix} y_{CMS} \\ y_{HMS} \\ y_{OMS} \\ y_{NMS} \end{bmatrix} = \begin{bmatrix} 1 - y_{PMS} \\ 0.5 n_{H,US} - 1.5 n_{N,US} - 0.5 y_{FMS} n_{H,FS} \\ 1 + 0.25 n_{H,US} - 0.5 n_{O,US} - 0.75 n_{N,US} - \left(1 + 0.25 n_{H,PS} - 0.5 n_{O,PS}\right) y_{PMS} \\ n_{N,US} \end{bmatrix}$$

Mineral yield coefficients shoot growth with $y_{NVS} = 0$ (i.e. $n_{N,US} = y_{VS} n_{N,VS}$)

$$\begin{bmatrix} y_{CVS} \\ y_{HVS} \\ y_{OVS} \\ y_{NVS} \end{bmatrix} = \begin{bmatrix} 1 - y_{VS} - y_{PVS} \\ 0.5\left(n_{H,US} - y_{VS} n_{H,VS} - y_{PVS} n_{H,PS}\right) \\ 1 + 0.25 n_{H,US} - 0.5 n_{O,US} - \left(1 + 0.25 n_{H,VS} - 0.5 n_{O,VS}\right) y_{VS} - \left(1 + 0.25 n_{H,PS} - 0.5 n_{O,PS}\right) y_{PVS} \\ 0 \end{bmatrix}$$

Mineral yield coefficients shoot maturity maintenance and development

$$\begin{bmatrix} y_{CDS} \\ y_{HDS} \\ y_{ODS} \\ y_{NDS} \end{bmatrix} = \begin{bmatrix} 1 \\ 0.5 n_{H,US} - 1.5 n_{N,US} \\ 1 + 0.25 n_{H,US} - 0.5 n_{O,US} - 0.75 n_{N,US} \\ n_{N,US} \end{bmatrix}$$

Mineral yield coefficients shoot reproduction

$$\begin{bmatrix} y_{CF} \\ y_{HF} \\ y_{OF} \\ y_{NF} \end{bmatrix} = \begin{bmatrix} 1 - y_F \\ 0.5 n_{H,US} - 1.5 n_{N,US} - \left(0.5 n_{H,F} - 1.5 n_{N,F}\right) y_F \\ 1 + 0.25 n_{H,US} - 0.5 n_{O,US} - 0.75 n_{N,US} - \left(1 + 0.25 n_{H,F} - 0.5 n_{O,F} - 0.75 n_{N,F}\right) y_F \\ n_{N,US} - y_F n_{N,F} \end{bmatrix}$$

Mineral coefficients nitrate to ammonia conversion

$$\begin{bmatrix} y_{CNoxR} \\ y_{HNoxR} \\ y_{ONoxR} \\ y_{NNoxR} \end{bmatrix} = \begin{bmatrix} 1 \\ 1 - y_{NoxA} \\ 1 - 2 y_{NoxA} \\ y_{NoxA} \end{bmatrix}$$

Mineral yield coefficients of N-rich and carbohydrate formation in roots: same as shoots

Mineral yield coefficients root catabolism

$$\begin{bmatrix} y_{CUR} \\ y_{HUR} \\ y_{OUR} \\ y_{NUR} \end{bmatrix} = \begin{bmatrix} 0 \\ 0.5n_{H,UR} - 0.5n_{H,N} - \left(0.833 - 0.5n_{H,N}\right) y_{UR} \\ 0 \\ 0 \end{bmatrix}$$

Mineral yield coefficients root maintenance

$$\begin{bmatrix} y_{CMR} \\ y_{HMR} \\ y_{OMR} \\ y_{NMR} \end{bmatrix} = \begin{bmatrix} 1 - y_{PMR} \\ 0.5n_{H,UR} - 1.5n_{N,UR} - 0.5y_{PMR}n_{H,PR} \\ 1 + 0.25n_{H,UR} - 0.5n_{O,UR} - 0.75n_{N,UR} - \left(1 + 0.25n_{H,PR} - 0.5n_{O,PR}\right) y_{PMR} \\ n_{N,UR} \end{bmatrix}$$

Mineral yield coefficients shoot growth with $y_{NVR} = 0$ (i.e. $n_{N,UR} = y_{VR}n_{N,VR}$)

$$\begin{bmatrix} y_{CVSR} \\ y_{HVR} \\ y_{OVR} \\ y_{NVR} \end{bmatrix} = \begin{bmatrix} 1 - y_{VR} - y_{PVR} \\ 0.5\left(n_{H,UR} - y_{VR}n_{H,VR} - y_{PVR}n_{H,PR}\right) \\ 1 + 0.25n_{H,UR} - 0.5n_{O,UR} - \left(1 + 0.25n_{H,VR} - 0.5n_{O,VR}\right) y_{VR} - \left(1 + 0.25n_{H,PR} - 0.5n_{O,PR}\right) y_{PVR} \\ 0 \end{bmatrix}$$

Mineral yield coefficients shoot maturity maintenance and development

$$\begin{bmatrix} y_{CDR} \\ y_{HDR} \\ y_{ODR} \\ y_{NDR} \end{bmatrix} = \begin{bmatrix} 1 \\ 0.5n_{H,UR} - 1.5n_{N,UR} \\ 1 + 0.25n_{H,UR} - 0.5n_{O,UR} - 0.75n_{N,UR} \\ n_{N,UR} \end{bmatrix}$$
